# A logic-based diagram of signalling pathways central to macrophage activation

**DOI:** 10.1186/1752-0509-2-36

**Published:** 2008-04-23

**Authors:** Sobia Raza, Kevin A Robertson, Paul A Lacaze, David Page, Anton J Enright, Peter Ghazal, Tom C Freeman

**Affiliations:** 1 Division of Pathway Medicine, University of Edinburgh, The Chancellor's Building, College of Medicine, 49 Little France Crescent, Edinburgh, EH16 4SB, UK; 2 Computation and Functional Genomics Laboratory, Sanger Institute, Wellcome Trust Genome Campus, Hinxton, Cambridge, CB10 1SA, UK; 3 Centre for Systems Biology, University of Edinburgh, Darwin Building, King's Building Campus, Mayfield Road, Edinburgh, EH9 3JU, UK

## Abstract

**Background:**

The complex yet flexible cellular response to pathogens is orchestrated by the interaction of multiple signalling and metabolic pathways. The molecular regulation of this response has been studied in great detail but comprehensive and unambiguous diagrams describing these events are generally unavailable. Four key signalling cascades triggered early-on in the innate immune response are the toll-like receptor, interferon, NF-κB and apoptotic pathways, which co-operate to defend cells against a given pathogen. However, these pathways are commonly viewed as separate entities rather than an integrated network of molecular interactions.

**Results:**

Here we describe the construction of a logically represented pathway diagram which attempts to integrate these four pathways central to innate immunity using a modified version of the Edinburgh Pathway Notation. The pathway map is available in a number of electronic formats and editing is supported by yEd graph editor software.

**Conclusion:**

The map presents a powerful visual aid for interpreting the available pathway interaction knowledge and underscores the valuable contribution well constructed pathway diagrams make to communicating large amounts of molecular interaction data. Furthermore, we discuss issues with the limitations and scalability of pathways presented in this fashion, explore options for automated layout of large pathway networks and demonstrate how such maps can aid the interpretation of functional studies.

## Background

The innate immune response is executed at the molecular level by a complex series of interwoven signalling pathways. In this context, pathways may be defined as a network of directional interactions between the components of a cell which orchestrate an appropriate shift in cellular activity in response to a specific biological input or event. Whilst our ability to perform quantitative and qualitative measurements on the cellular components has increased massively in recent years, as has our knowledge on how they interact with each other, we still struggle to translate these observations into graphical and computationally tractable models. However without such models we can not hope to truly understand biology at a systems level.

Traditionally, representations of molecular pathways have been produced *ad hoc *and frequently included in reviews and original papers. Whilst they are clearly useful aids to understanding cellular events, even at their best, they are not sufficient by themselves, relying on extensive textual descriptions to explain what is shown pictorially. Recent years have seen considerable growth in the availability of public and commercial databases offering searchable access to pathways and interaction data derived from a combination of manual and automated (text mining) extraction of primary literature, reviews and large-scale molecular interaction studies. Using these tools it is possible to view a range of canonical pathway views or generate networks of interactions based on a given query. However, all of these efforts are let down by one or a number of key factors. The notation used in diagrams to depict one molecule's interaction with another is varied, often ambiguous and therefore limited in its ability to depict the exact nature of the relationship between components of a pathway. There is often a lack of direct access to the experimental evidence relating to the interactions depicted or to the dataset as a whole. Similarly, labelling of the pathway components often uses non-standard nomenclature or mixes protein names from one species with that of another, such that again the reader is left uncertain as to what exactly is being shown. Finally, pathway diagrams usually focus only on a small part of a biological system and one which often reflects the curator's bias, such that the 'same' pathway described by different individuals may share little in common. Whatever the source of these pathways and networks they generally suffer from graphically poor representation with ambiguity around the precise identity of what is being shown and the exact nature of their interaction. In order to address these issues the groups of Kohn and Kitano began to devise new approaches to pathway notation using many ideas adopted from the electronics industry [1-3]. In particular the MIM (molecular interaction map) notation [3] a form of entity-relationship representation and the process description notation (PDN) [1], respectively. Since then there has been an increasing interest in the systems biology community to develop a consensus view on a standard approach for representing biological pathways [4]. Whilst this process is now well advanced there is currently no internationally agreed standard graphical notation system for building pathway diagrams and a paucity of worked examples of this type of notation in use. Examples of pathways that have been published using these notation systems include a molecular interaction map of macrophage signalling [5] and Toll-Like-Receptor signalling [6] which have been depicted using the PDN scheme and cell cycle control and DNA repair presented in the MIM notation [2].

Over the last four years we have been developing a notation scheme for the depiction of biological pathways that borrows many of the ideas of existing notation systems but attempts to address some of their short comings. The Edinburgh Pathway Notation [26] uses a logical state-transition representation to describe biological pathways, similar to PDN. The work described here follows on from this initial publication and reports a modified version of the EPN scheme which is aligned with the developing international SBGN standard but has a number of important differences with the scheme as currently proposed. Crucially, the notation provides a logical context for interactions between components in the pathway, it can display the temporal order of reactions and can be mapped to the machine-readable SBML (systems biology markup language) [7]. Of primary importance to this notation scheme and indeed the SBGN is the desire to develop pathway maps that are 'readable' by a biologist. Since the pathway maps are primarily produced as a tool for communication it is critical that they are easily understandable and the notation can be applied and read by biologists with minimal training. Other objectives (of the SBGN) are that the notation should be computable, compact, show sub-cellular localization and be tolerable of incomplete knowledge. Whilst all of these objectives are valid, fulfilling them in practice is far from trivial and there are few worked examples of large pathway diagrams depicted in standard notations, available in the public domain.

The innate immune response is orchestrated by series of signalling pathways that have evolved to elicit an appropriate defensive response to attack by pathogenic organisms. Pathogen sensing involves pattern recognition receptors such as the toll-like receptors (TLR's) which in mammalian cells constitutes a family of up to 11 [8] transmembrane receptors each responsible for distinguishing particular pathogen-associated molecular patterns (PAMPs). Detection of pathogen molecules by these receptors results in the recruitment of various adaptor proteins and the activation of downstream signal transduction cascades [9,10]. Activation of *de novo *gene expression follows, which ultimately acts to recruit new proteins and augment the response to infection. Interferons (IFNs) are central to this response, as are (amongst others) interferon regulatory factors (IRFs), JAK/STAT signalling proteins and the nuclear factor-kappa B (NF-κB) family of proteins [11]. The IRF family of transcription factors bind specific DNA sequences, as do the STAT proteins, present on the promoter of target genes [12,13]. NF-κB signalling can regulate transcription through a combination of NF-κB protein homo- and heterodimers [14-17]. These pathways are also known to regulate components of the apoptotic pathway, thereby providing the potential for cells to undergo a programmed cell death [18], the ultimate cellular sacrifice in defence of the organism.

The TLR, IFN, NF-κB and apoptosis pathways are of central importance in defining the macrophages response to pathogens and do so in a highly inter-dependant manner [11]. Extensive literature describing the pathways and their interconnectivity, like so many others in biology, is available but only from multiple and disparate sources. In our effort to understand these events as a basis for interpreting analyses of host-pathogen interactions and the inflammatory response in the macrophage, we have endeavoured to construct an integrated and logic-based pathway diagram of signalling cascades fundamental to macrophage activation using a current version of the EPN scheme. We present the results of these labours as an example of our on going work in this area and hope that this map will be used to supplement and contrast with the efforts of others [6] in this area.

## Methods

### Collection of Molecular Interaction Data and Biological Representation of Pathways

In an effort to describe and consolidate knowledge of pathways central to macrophage activation we have constructed a pathway diagram based on published literature. Ideally, two published papers citing protein-protein or protein-gene interactions were required for the inclusion of a given interaction on to the pathway diagram. In some circumstances we accepted one piece of published evidence if the paper described extensive experimental verification of the interaction. This was deemed necessary as two publications per interaction can limit inclusion of potentially interesting interactions included in other pathway resources (KEGG, Reactome etc) and newly discovered interactions. It is also important to note that the primary task of this exercise was to develop a 'consensus' of knowledge and information about a given pathway.

A list of interactions to be mapped was compiled [see Additional file 1], including details about the nature of the interaction and source of the information. A pathway map was then drawn using the principles laid down by the EPN scheme. These include the concept that the molecular components of a pathway be they proteins, protein complexes and genes (or in principle any other cellular component that plays a part in a pathway) are represented as simple shapes containing a unique and unambiguous identifying label. Attempts to depict pictorially the functional activity or functional domains of components have been avoided as this adds to the visual complexity of the diagram and can be misleading. For consistency components (nodes) have been named by their official human genome nomenclature (HGNC) symbol, although in certain instances we have felt it necessary due to the wide-spread use of other naming conventions to supplement this with additional annotation. For example we have used the name tBID to differentiate the truncated (active) form of the protein from its precursor (BID) and similarly in order to distinguish the native (inactive) form of caspases we used the suffix Pro e.g. ProCASP3 from the active cleaved form (CASP3). We have also included additional naming conventions to differentiate between protein forms e.g. in the NF-κB pathway (p50, p52 etc) or included common aliases where they are prevalent in the literature, these names being placed in brackets after the official name. Whilst the use of such *ad hoc *naming conventions is in theory undesirable, they are still in common use and alternative ways to differentiate between protein forms is not supported under the HGNC and standard naming conventions for describing proteins in their various modified forms (truncated, cleaved, activated by cleavage etc) does not yet exist. Where pathway components are protein complexes, the name of the complex is given as a concatenation of the names of its constituent parts, although this has in some cases been supplemented by the inclusion of common names such as 'apoptosome' to denote the complex between CASP9, CYCS and APAF1. Components are depicted at the site of their activity and are shown only once in any given cellular compartment unless different activation states of the components are known due to phosphorylation, ubquitinisation, cleavage etc., when these molecular states may be shown as connected but individual entities. The state of a component may be shown as a supplement to the components name e.g. active [A], inactive [I], phosphorylated [P]. Interactions (edges) between components or transitions between one cellular compartment and another, are shown as arrows which either contact interacting partners via Boolean logic operators (&, OR, NOT) and/or transition/annotation nodes that provide information as to the nature of the interaction or transition from one state to another. Attempts to depict molecular details of interactions and state transitions such as the exact site of a protein's phosphorylation, have generally been avoided. Whilst important, if depicted on a map of this size the information quickly clutters up the diagram rendering it inaccessible to the casual reader. However, in cases where such details are necessary to differentiate one component form from another they should be added. Finally, layout of the elements and interactions that make up the pathway should be such that it is relatively easy to follow the direction and nature of flow of information from the initial trigger to the eventual outcome. In an effort to achieve this, where possible interacting map components are drawn close together keeping edge lengths short and easy to follow, crossover of edges is kept to a minimum and every effort is taken to keep connecting edges separate, with a minimum number of changes in direction to get from one point to another.

**Figure 1:**
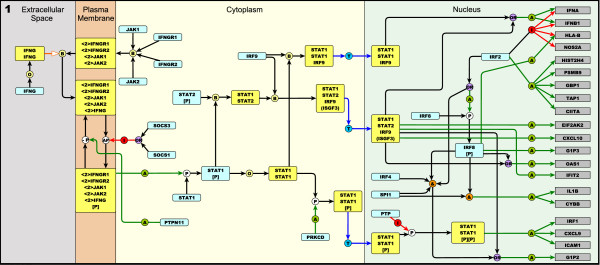
**Manual layout of Type II interferon signalling (IFNG) taken from the integrated pathway diagram (Figure 2)**. The pathway is arranged to flow from left to right. Components are coloured according to type (protein, complex or gene) and arranged within the sub-cellular compartments in which they are active. This pathway is initiated by IFNG binding to its receptor and a subsequent phosphorylation cascade involving a number of the JAK and STAT family of proteins. Several transcriptionally active complexes are formed (STAT1 homodimer, ISGF3 complex, STAT1:STAT1:IRF9 complex) and the pathway culminates with the transcriptional activation of target genes.

**Figure 2:**
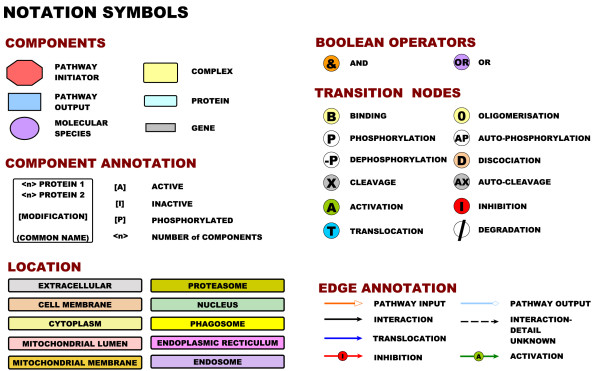
**Symbols of the modified Edinburgh Pathway Notation**. Unique shapes and identifiers are used to distinguish between each element of the notation allowing its interpretation even in the absence of colour. Colour maybe used for aesthetic purposes and to ease identification of nodes. The notation can be broadly divided into four categories; components, boolean operators, transition nodes and annotated edges. Components consist of any interacting species from proteins, complexes, genes or other molecular species (pathogens, DNA, RNA). Pathway initiators are also presented in the notation. Boolean operators are essential for capturing the dependencies of an interaction. Transition nodes provide information as to the nature of the interaction (such as cleavage, translocation, phosphorylation). Edges are directional and can be coloured for visual impact. Distinctive arrow-heads are used to distinguish between the pathway inputs and outputs but are otherwise avoided. Instead in-line edge annotation is used to add a visual cue as to the meaning of an edge. Cellular compartmental information is provided by physical location and backdrop or by colouring nodes according to their sub-cellular location.

The pathway map was drawn using the freely available program yEd graph editor (yFiles software, Tubingen, Germany). yEd is a general purpose graphical tool designed for the depiction of networks. Although not specifically designed for biological pathway depiction it has been used previously for this and similar purposes [19,20] and has a range of characteristics and capabilities that make it ideally suited for the job. Initially pathways were laid out by hand. Areas of the canvas were defined as representing specific compartments of the cell e.g. plasma membrane, cytoplasm, nucleus etc., and cellular components and the interactions in which they took part were drawn in the appropriate space. A section of the overall map describing IFNG receptor signalling laid out according to the cellular location of the components has been included as an example of the notation scheme in action (Figure 1) and a list of notation symbols used here is provided in Figure 2.

**Figure 3:**
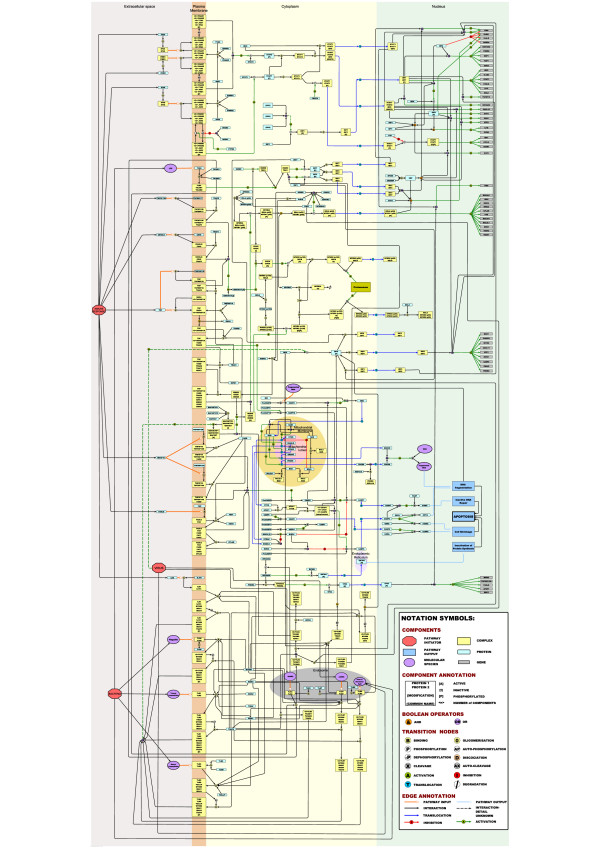
**Integrated pathway map of signalling in the macrophage**. The diagram includes the interferon signalling, NF-κB, apoptosis and toll-like receptor pathways, all represented as one integrated pathway due to their overlapping interactions. In general interactions of the interferon response pathway are in the top quarter of the map, with NF-κB directly below. Apoptosis is presented halfway down the map and toll-like receptor signalling is in the bottom quarter. 154 different protein or gene nodes are included in the pathway, along with 80 different complexes and 12 other molecular species (such as pathogens, DNA, RNA). The pathway diagram represents 272 different interactions.

The combined map of macrophage activation pathways described here (Figure 3) is available for download [21] and presented in a number of image (.jpeg, .pdf) and graphical formats (.xml, .graphml). The .graphml file [see Additional file 2] can be opened in yEd graph editor [22] and in this format is available for editing or expansion. PubMed IDs supporting the interactions of the pathway are stored on appropriate edges within the .graphml version of the diagram. We have found the yEd program to be relatively intuitive to use and to require minimal or no training. Hence the pathway diagram presented here is easily accessible, distributable and can be modified by end users to suit their interests or knowledge-base. The EPN can be mapped to SBML and we are in the process of creating a SBML version of the map described here.

**Figure 4:**
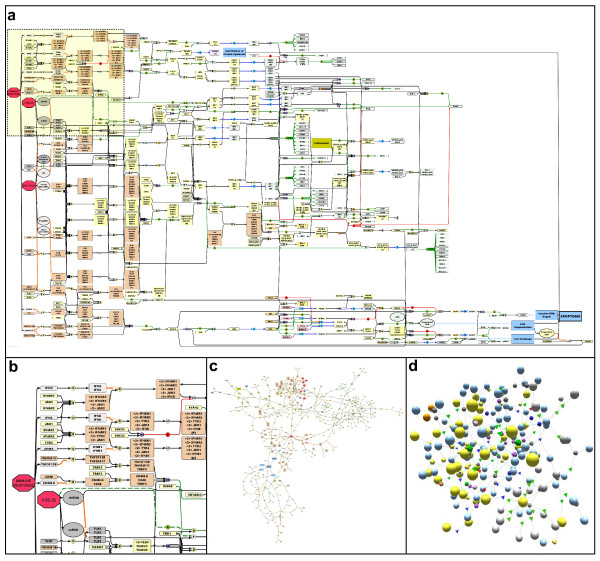
**Automated layouts of the pathway diagram**. (4a) a hierarchical-classic layout was applied to the entire pathway and the orientation was set to flow from left to right. Nodes are coloured according to their sub-cellular location. With this layout the flow of pathway information and biological logic is maintained, such that the inputs to pathway are placed at the left side of the diagram and these can be followed through to the outputs at the right hand side. (4b) a detailed inset of the hierarchical-classic layout of the integrated pathway taken from 4a. (4c) Organic-classic automated layout of the entire pathway generated in yEd graph editor. Although the directionality of flow in the pathway is lost, interacting partners tend to be placed in close proximity of each other in this layout. (4d) a 3-dimentional network of the apoptosis interactions in the pathway generated using BioLayout Express^3D^. This network can be queried for pathway information. Unique shapes are used to identify the different pathway notation symbols; spheres denote interacting components (proteins, genes, complexes), decahedron shapes represent boolean operators or transition nodes and tetrahedron shapes correspond to the in-line edge annotation (in this case activation, or inhibition). All notation symbols are coloured to correspond to the colour scheme applied in the 2-dimentional pathway diagram (e.g. complexes are yellow, proteins are blue, and activation-annotations are green). Furthermore interacting components are sized according to type, such that spheres representing complexes appear larger than proteins or genes. As with the 2-dimentional diagram the colour scheme used is customisable. The 3-dimentional network retains the information captured in the 2-dimentional pathway and although spatial placement of nodes in relation to their sub-cellular location has been lost, this information can be retrieved by querying the network and/or colouring nodes according to their sub-cellular location.

As the complexity of maps increases and the interactions between components become evermore intertwined, manual organization of these events becomes time-consuming and difficult. However, the pathway diagrams have been specifically drawn as directional networks. As such layout of the pathway maps can be aided by use of various automated layout algorithms. The hierarchical layout and classic-orthogonal edge routing applications within the yEd software were the most effective in terms of providing an easily interpretable view of directional flow in the diagram (Figure 4a, b). However, other layout algorithms such as the organic layout function (Figure 4c) can also provide different views of the pathway. Furthermore this pathway can be easily converted into a 3-D network (Figure 4d) in BioLayout *Express*^3D ^[23]. Whilst 3-D pathway networks do not readily support user readability of the interactions, it provides an environment where very large graphs may be plotted (15,000 nodes, 2.5 million edges) and queried. As such these tools can aid interpretation of the innate structure within the network of interactions of large pathway diagrams and together provide a solution, albeit not necessarily a perfect one, to the issue of scalability. With these capabilities it will be possible to scale up these diagrams to the point where they may contain thousands of components, operators and transition nodes.

### Network Analysis of the Transcriptional Response of Mouse Bone Marrow Derived Macrophages to Interferon-gamma Treatment

Primary mouse bone marrow derived monocytes were prepared from male balb/c mice 10–12 weeks old. Cells were washed, resuspended in DMEM-F12/10% FCS/L929 medium and counted before being plated in a 24-well plate at a concentration of 5 × 10^5 ^cells/well. To differentiate the cells from monocytes into primary macrophages, cells were then incubated for 7 days in DMEM-F12 growth media supplemented with 10% L929 cell suspension releasing the MCP-1 macrophage stimulating factor, with media changes on days 3 and 5. On day 7 the growth medium was replaced with DMEM-12/10%FCS medium containing 10 u/ml recombinant mouse interferon-gamma (Pierce-Thermofisher Scientific, Rockford US) and harvested 1, 2, 4 & 8 h following treatment or collected pre-treatment (0 h). Total RNA was harvested from the cells using an RNeasy Plus kit (Qiagen) according to manufacturer's instructions. RNA was quantified and quality controlled using a NanoDrop spectrophotometer (NanoDrop Technologies) and BioAnalyser 2100 (Agilent). Replicate 150 ng samples of total RNA derived from two separate wells per time point were labelled using the Affymetrix whole transcript labelling protocol and hybridized for 16 h at 45°C to Affymetrix mouse exon 1.0 ST arrays. They were then washed and scanned according to manufacturer's recommendations.

**Figure 5:**
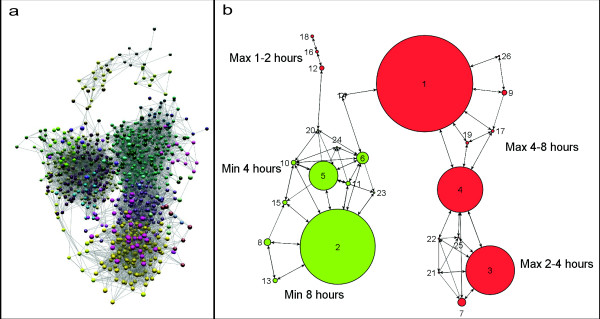
**a) A network graph of differentially expressed genes following Ifng treatment**. A Pearson correlation cut-off of 0.9 was set to filter edges in the network and the resultant graph was clustered using the graph-based clustering algorithm MCL set at an inflation value of 2.2. Each node represents a transcript and nodes are coloured according to the cluster to which they belong. Nodes belonging to the same cluster share a common pattern of expression over the time-course following Ifng treatment. **b) A view of the 26 clusters defined from the network graph in 5a**. The size of each sphere representing a cluster corresponds to the size of its node membership. Clusters are assigned a description of the co-expression pattern they present over the time course and are coloured according to whether the nodes within those clusters are up-regulated (orange) or down-regulated (green) following the Ifng treatment.

Data (ArrayExpress Ac. No: E-MEXP-1490) was normalized using the RMA package within the Affymetrix Expression Console software and annotated. Transcripts which might be considered to be differentially expressed were identified using either the Empirical Bayes function within Bioconductor [24] or using the annova function within GeneSpring (Agilent Technologies, Stockport, Cheshire) with a 1.6 fold cut-off. In total 1,678 transcripts were identified by one or both of these filters. The data corresponding to this list was then loaded into the network visualization tool BioLayout Express^3D ^[23] using a Pearson correlation cut-off of 0.9 to filter edges. The resultant network graph (Figure 5a) of 1,491 nodes was clustered using the graph-based clustering algorithm MCL [25] set at an inflation value of 2.2 resulting in 26 clusters (Figure 5a & b). Clusters composed of transcripts that were up-regulated were then further collated into 3 groups; genes up-regulated at (1) 1–2 hours, (2) 2–4 hours and (3) 4–8 hours post-treatment and genes that were both differently expressed and present on the integrated pathway diagram were highlighted on the map.

## Results and Discussion

We set out to use the EPN scheme as originally published [26]. However, during construction of the maps described here the notation system was found to be too limiting to convey certain biological concepts and overly complicated for others. A simplification of certain aspects of the notation was therefore deemed necessary in order to achieve the objectives outlined above, in particular human readability. Modifications made to the EPN were not intended to change the built in logic of the notation scheme but rather merely enhance the visual characteristics of the diagrams produced. One of the major modifications we have made is in the reliance of the original EPN (and the emerging SBGN standard) on multiple types of arrow heads to infer different meaning to the interactions. We have used only one type of arrowhead and relied far more heavily on the use of transition nodes or annotation nodes to infer the nature of the transition from one molecular state to another and add information to edges. We found this system to improve the readability of the maps as well as provide greater flexibility in the range of concepts that may be depicted. The pathway diagrams created using this notation scheme function without the use of colour and do not therefore lose their semantics if viewed without it. Nevertheless, colour does provide a powerful device for increasing the visual impact of the figure. Here we have generally chosen apposite or symbolic colours to represent the appropriate interaction; for example red for inhibition, green for activation. However, it must be emphasized that the exact colour scheme is not important and should be seen as customizable to suit an individuals taste or limitations in colour recognition.

The pathway map described here (Figure 3) consists of a total of 295 nodes of which 140 are proteins, 99 complexes, 44 genes, and 12 other components (pathogens, DNA, RNA etc). A total of 272 interactions are described in the pathway map, of these 85 are binding events, 149 are various activation state modulations (67 activation of gene expression, 26 phosphorylation, 7 auto-phosphorylation, 1 dephosphoylation, 23 cleavage, 9 translocations and 16 activation by processes not defined). There are 10 inhibition reactions, 4 of these are inhibition of gene expression, 3 are inhibition of cleavage, and 1 is an inhibition of translocation. A total of 26 translocation events occur as well as 2 protein dissociations. 282 different references support the interactions shown on the pathway [see Additional file 3]. In many circumstances the same paper may describe multiple interactions, for example Chaudhary *et al*., (1997) report that both TNFRSF10A and TNFRSF10B recruit the protein FADD during apoptosis signalling [27]. A detailed description of the biological content of this pathway diagram is given in Additional file 4.

**Figure 6:**
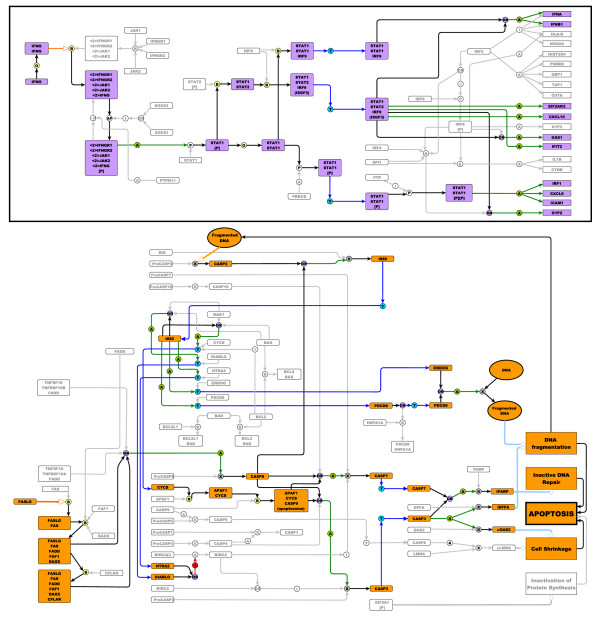
**Follow through of signalling pathways stimulated by IFNG (6a) and FASLG (6b)**. The signalling events following the input signals of IFNG and FASLG have been highlighted on the entire map in lilac and orange, respectively. The nodes activated or directly affected by FASLG or IFN-gamma binding to their receptors are coloured and the interaction edges and gates are also highlighted. Nodes and edges not directly downstream of the FASLG or IFNG signalling are shown in grey. This figure demonstrates inputs into the pathway can clearly be followed to the expected outcome events. In the case of IFNG-input, gene transcription is the resulting event, and in the case of FASLG, apoptosis. Furthermore these examples clearly depict the interactions of the pathway can be followed logically and do not result in unexpected crosstalk.

In order to check the integrity of the network each input (e.g. cytokine or pathogen molecule), was highlighted in turn and the logical flow of information from this input followed through the diagram. By following the flow of information from each pathway input, a different but expected output was observed, be that the activation of transcription or a process such as apoptosis (Figure 6a & b). This suggests that although several signalling pathways have been integrated to form this diagram the specificity of connectivity has not been lost.

### Use of Pathway Diagram in the Interpretation of Transcriptomics Data

**Figure 7:**
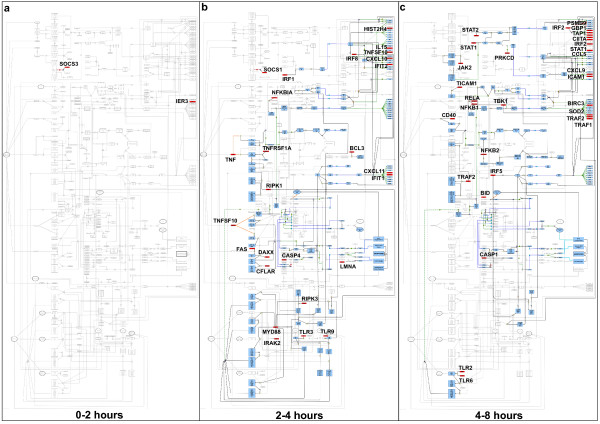
**The integrated pathway diagram presented at (a) 1–2 hours, (b) 2–4 hours and (c) 4–8 hours post-Ifng treatment**. Differentially expressed genes are highlighted in red and the possible consequential downstream events resulting from the changes, (assuming de novo protein synthesis) are highlighted in blue.

In order to demonstrate the utility of this pathway diagram in the interpretation of transcriptomics data we have examined the transcriptional events following the treatment of mouse bone marrow derived macrophages (BMDM) with interferon-gamma (Ifng). Using the network analysis tool BioLayout *Express*^3D ^[23] we constructed a 3-D network of transcripts identified as being differently expressed following Ifng stimulation (Figure 5a). 1,491 transcripts were represented within the network, 1,274 of which grouped into 26 clusters with ≥ 5 members (Figure 5b). There are 154 unique proteins/genes represented on the pathway map, 55 of which are represented within these clusters and a further 3 components were in the transcriptional network but did not fall into a cluster [see Additional file 5]. All of the genes represented on the map were in clusters of up-regulated genes. Clusters of transcripts representing genes activated at different times following treatment were then further collated into 3 groups of up-regulated genes; genes activated at (1) 1–2 hours, (2) 2–4 hours and (3) 4–8 hours post-treatment. Genes that were activated and included in the set of mapped genes were then highlighted on the map and the possible downstream consequences (assuming *de novo *protein synthesis and activity following an increase in gene transcription) were highlighted (Figure 7). In this way is has been possible for the first time to interpret these transcriptional events in the context of the possible consequences of these observations.

During the very early phase (0–2 hours) of the response to Ifng treatment only two genes, SOC3 and IER3, corresponded to pathway components shown in the diagram (Figure 7a). SOCS3 (suppressor of cytokine signalling 3) is an inhibitory protein of Interferon-gamma receptor complex signalling and has also been reported elsewhere to be expressed in macrophages following interferon treatment [28]. The up-regulation of SOCS3 represents a classical negative feedback loop required to regulate the magnitude and duration of signalling downstream of the IFNG receptor signalling, in addition to limiting the response to any subsequent cytokine stimulus [29,30]. IER3 (immediate early response 3) a stress inducible gene is a target gene of the NF-κB signalling complex NFKB1-RELA [31] and is known to be activated in response to a variety of cellular stress signals [32-35]. Although IER3 is not depicted to be directly induced by Jak-Stat signalling we understand that connectivity exists between this signalling system and the NF-κB pathway. 25 components of the pathway diagram were also regulated 2–4 hours post-Ifng treatment (Figure 7b). Most noticeably members of apoptosis and TLR signalling were changing during this time and interestingly these changes occurred around the initiation or receptor signalling region of these pathways. When observed in more detail we identified that three potential mechanisms of apoptosis induction were targeted; TNF, TNFRSF10 and FAS signalling. TNF, its receptor TNFRSF1A and an adaptor protein RIPK1 are all up-regulated, as is TNFSF10 (Trail-ligand). FAS and adaptor molecules (DAXX and CFLAR) of the FAS receptor were also increased in their expression. A similar observation was also made for TLR-signalling, as a number of key adaptors proteins (including MYD88 and IRAK2) were up-regulated in the 2–4 hour timeframe. By activating the TLR system and apoptotic machinery the cells appear to preparing themselves for contact with pathogens and priming themselves for apoptosis. One possible consequence of TLR signalling when followed though on the pathway diagram is the activation of the IRF5 transcription factor and indeed 5 targets of IRF5 were up-regulated at the 2–4 hour time phase (*CXCL11, IFIT1, CXCL10, IFIT2*, and *TNFSF10*). Moreover IRF5 was itself regulated at the later time points (4–8 hours) post-IFNG treatment. Another consequence of TLR-signalling is the activation of the NF-κB pathway and again the key constituents of this pathway (NFKB1 and RELA) were activated at the later time points as were some transcriptional targets of this complex. During the 4–8 hours period BID, an important amplifier of apoptotic input signals via the mitochondrial apoptotic pathway, was up-regulated (Figure 7c). BID can be cleaved and activated by any of the three aforementioned apoptotic mechanisms (FASLG, TNFSF10 and TNF) [36-38] that were altered during the earlier time phase. Also up during the latter hours were members of the Jak-Stat pathway (JAK2, STAT1, STAT2, and PRKCD which phosphorylates and activates STAT1) and some target genes of the Jak-Stat pathway, which could represent increased sensitivity to IFN or other cytokine signalling.

We are acutely aware that the current pathway diagram covers only a relatively small number of the genes shown to be transcriptionally regulated following Ifng treatment. For instance none of the genes shown to be down-regulated by Ifng are shown in the diagram. However even with the current limited coverage we have been able to extrapolate some interesting observations by visualizing the changes and the possible downstream effect of the changes. It has been possible to appreciate the connectivity and co-dependency of the changes over time and using this approach the detail of how signalling in one region may have downstream effects on another signalling system can be hypothesized and in many examples here extracted.

### Critical Review of Pathway

In constructing this integrated map of macrophage activation pathways we have attempted to represent events in a detailed, accurate and logical fashion. However, it must be emphasized that this map is by its nature a biased view of events. Its construction has been primarily driven by our interest in understanding signalling events in the macrophage and interpretation of the literature is an unavoidably flawed process; determining what constitutes good evidence for an interaction and what does not, is often difficult to judge especially for those who do not specifically work in the area. Furthermore, any view of what constitutes a given pathway is also highly subjective and is always being driven by an individual's perspective and scientific trends, as well as current knowledge. Even though pathway diagrams typically depict individual pathways in isolation of other systems, in reality it is well recognized that there is significant overlap in pathway membership and cross-talk between related pathways. Input from one signalling pathway can influence the outcome in another, underscoring the need to view the connections between various signalling systems. Indeed, when one searches for the known interactions of any well characterized protein using database tools such as String [39] or Ingenuity [40] one is potentially led in many directions, each interacting protein in turn leading to an ever expanding network of molecular interactions. Therefore when drawing pathway maps such as the one described here, it is impossible to include all the known interactions of any given component. We are aware there are other systems important in their regulation which have could be included, most noticeably, NOD/NALP receptor signalling, MAP kinase cascades, interleukin and other cytokine/chemokine systems, many aspects of the TNF-family of proteins, antigen presentation and cell cycle pathway, to name but a few. Some of these systems are now being added to the pathway diagram but this is largely being driven by our need to interpret the results of systems-level analysis of the macrophages response to pathogens and cytokines. Indeed, the fact that this pathway is far from complete is further emphasized by its use in interpreting the transcriptional response to Ifng. Of the 1,141 transcripts falling into clusters of co-regulated genes following Ifng treatment, only 55 were represented on the map and the map so far includes only 44 genes in total as being regulated by any transcription factor. This therefore highlights the fact that there is some considerable way to go if we are to generate a complete model of the potential downstream events following the interferon signalling cascade.

In the case of the signalling systems described here, the interaction data is derived from the available literature and is therefore dependant on the quality of that work, the biological system from which that information was derived and as already mentioned represents a subjective view of the information available. Seldom do signalling pathways operate independently of each other therefore analyzing only a subset of nodes known to belong to a particular pathway is unlikely to be insightful as to the activity of the system as a whole. With so many of pieces of the jigsaw missing and many aspects of the activity of these large integrated molecular networks still unknown, performing meaningful analyses on relatively small sections of what is otherwise an immense network of interacting proteins, is unlikely to deliver accurate or biologically representative predictions for some time.

The current notation system used for the pathway presented here arguably works well up to this size of pathway and the end result we hope will serve as a useful reference for biologists interested in these systems. However, scalability of pathway diagrams is an important issue especially when a compromise must be reached between presenting a human readable map with one that captures the extensive interaction data now available for many molecules. Although we intend to continue to consolidate and add interactions to the current map we are aware that this could prove difficult in number of respects. When new components are added, in order to place them near to the site of their interacting partners the layout of the entire graph sometimes needs to be manually altered to make space. Furthermore, as functional units of an integrated pathway network frequently share components, proteins often referred to as hubs, it is often impossible to place a component near to all its interacting partners requiring edges (interactions) to span large distances across the map. One method of reducing long edge lengths is to depict individual components more than once within a given cellular compartment. However, this in turn adds to the issue of scalability as the additional nodes consume more space, add more complexity and the visual link between components of the pathway are lost. We have therefore been exploring alternative approaches to overcome the issue of scalability in pathway depictions. One approach is to use automated layout algorithms to draw the relationships between pathway components. Certain layout algorithms are very effective at displaying connectivity between components with little or no need for manual intervention (Figure 4a & b). This allows the rendering of relatively large pathway diagrams quickly and easily, whilst retaining much of the biologist friendly aspects to the diagrams. What is lost is the spatial layout according to the cellular compartment of components. However this aspect can be retained, at least in part, by the use of colour to signify in which compartment they reside. A second approach for dealing with large interaction networks/pathways is to visualize them in 3-dimensional space. Using the tool BioLayout *Express*^3D ^recently developed by us [23] we have found it possible to render very large networks. In this instance the shape, size and colour can all be used to distinguish between different component types and colour can be overlaid to indicate cellular compartment (Figure 4d). Whilst arrow heads are not supported in 3-D mode directionality is reinstated when graphs or selected portions of large graphs are converted to 2-D networks.

## Conclusion

With the majority of the components of life defined, at least at some level, there is an increasing desire to put the parts together in order to construct models of biological systems which can be tested and refined. In this respect, the value of logically presented pathway diagrams is becoming ever more apparent given the growing need to systematically organize and describe the interactions between the various components that make up a cell. Pathway diagrams serve several purposes; they can be used to capture a large amount of information, provide a point of reference for researchers with an interest in the pathway or particular member of that pathway, and can be used to aid the interpretation of systems level analyses. The pathway presented here is by no means a comprehensive view of all the pathways involved in macrophage activation, but acts a worked example of how a number of key pathways might be represented in what we hope is a logical and unambiguous fashion. However, with the visual modifications to the EPN scheme we believe we have fulfilled the primary objectives of providing a graphical notation that is both useable by biologists and which could still serve as the basis for computational model development. So whilst others have gone some way to address the issue of human readability of their pathway diagrams we believe that we have derived an elegant yet simple notation scheme that better addresses the needs of biologists. The mapping process is a continuing effort and during the next steps we aim to consolidate and expand the content of the diagram. This in turn may require refinements to the notation system as issues in depicting the relation between components and the cellular components in which they are active arise. As we enhance our understanding of individual signalling pathways and how they integrate with others this will aid understanding of immunological disorders at a molecular level. Building pathway diagrams or networks of interactions from the existing knowledgebase is one of the milestones towards the application of pathway and systems biology to the field of medicine.

## Authors' contributions

SR constructed the integrated pathway diagram, contributed to the development of the notation system, participated in the expression profiling and analysis, and helped to draft the manuscript. KR contributed to the pathway development efforts and standardisation of pathway data collection and storage. PL contributed to the construction of pathway, collection of molecular interaction data and development of the notation scheme. DP set up the Ifng time course study. AE developed BioLayout Express^3D ^visualisation of the pathway diagram. PG originally conceived the EPN scheme and has supported its continued development. TF oversaw and contributed to the pathway construction, orchestrated the development of the EPN scheme, conceived the Ifng time course study, and its analysis and drafted the manuscript.

## Supplementary Material

Additional file 1 Pathway interaction list. Interactions included in the pathway map are listed (in no particular order) in this data file. Official HGNC (human gene nomenclature committee) gene symbols are used to name the interacting components along with a brief description of the type of interaction and its cellular location. Entrez gene IDs of interacting components are also provided as are the PubMed IDs of the reference(s) supporting each interaction. Click here for file

Additional file 2 Diagram of signalling pathways central to macrophage activation. This file can be opened, viewed and edited by users using the freely available graph-editor software yEd (yFiles software, Tubingen, Germany). This can be downloaded at [22] where full downloading instructions are described. PubMed IDs supporting the interactions of the pathway are stored on appropriate edges within this .graphml yEd file. Once an edge is selected the PubMed ID may be viewed within the descriptions tab of the properties box for that edge. Click here for file

Additional file 3 Bibliography of references supporting interactions on the integrated pathway diagram. Within this document a list of the 282 different references supporting the interactions on the pathway map are provided in alphabetical order (by author name). Click here for file

Additional file 4 Description of the biological content of the pathway. A description of the signalling of the four pathways (Toll-like receptor, interferon, NF-κB and apoptosis) depicted on the integrated pathway diagram is provided here. The interconnectivity of these pathways and their significance in innate immune signalling is also discussed in this section. Click here for file

Additional file 5 Interferon gamma regulated genes. A summary of the analysis of the 58 genes present on both the pathway map and in a transcriptional network of differentially expressed genes following Ifng stimulation. A transcriptional network of all differentially expressed genes (above 1.6 fold change) was constructed and clustered using the graph-based clustering algorithm MCL set at an inflation value of 2.2. This resulted in 26 different clusters, which were then assigned a description of the co-expression pattern they represent over the time course. The cluster numbers and the descriptions of co-expression pattern are shown in this data sheet for the genes present on the pathway diagram. 3 of these genes did not appear in any cluster. Also included in this table is a summary of the gene expression changes according to annova and Empirical Bayes calculations. RMA normalized expression values are included for each gene across the time course as are gene descriptions and GO (gene ontology) annotations for the 58 genes. Click here for file
