# Supplementary material for: A logic-based diagram of signalling pathways central to macrophage activation
Source: BMC Syst Biol. 2008 Apr 23;2:36. doi: 10.1186/1752-0509-2-36 (PMC2383880; doi:10.1186/1752-0509-2-36)
Supplement: Additional file 3 — Bibliography of references supporting interactions on the integrated pathway diagram. Within this document a list of the 282 different references supporting the interactions on the pathway map are provided in alphabetical order (by author name). [file 1752-0509-2-36-S3.doc]

Bibliography of references supporting interactions on the integrated pathway diagram

1. Aggarwal BB: **Signalling pathways of the TNF superfamily: a double-edged sword**. *Nat Rev Immunol* 2003, **3**(9):745-756.

2. Aguet M, Dembic Z, Merlin G: **Molecular cloning and expression of the human interferon-gamma receptor**. *Cell* 1988, **55**(2):273-280.

3. Arlt A, Grobe O, Sieke A, Kruse ML, Folsch UR, Schmidt WE, Schafer H: **Expression of the NF-kappa B target gene IEX-1 (p22/PRG1) does not prevent cell death but instead triggers apoptosis in Hela cells**. *Oncogene* 2001, **20**(1):69-76.

4. Arnoult D, Gaume B, Karbowski M, Sharpe JC, Cecconi F, Youle RJ: **Mitochondrial release of AIF and EndoG requires caspase activation downstream of Bax/Bak-mediated permeabilization**. *Embo J* 2003, **22**(17):4385-4399.

5. Au WC, Yeow WS, Pitha PM: **Analysis of functional domains of interferon regulatory factor 7 and its association with IRF-3**. *Virology* 2001, **280**(2):273-282.

6. Bach EA, Tanner JW, Marsters S, Ashkenazi A, Aguet M, Shaw AS, Schreiber RD: **Ligand-induced assembly and activation of the gamma interferon receptor in intact cells**. *Mol Cell Biol* 1996, **16**(6):3214-3221.

7. Baldwin AS, Jr.: **The NF-kappa B and I kappa B proteins: new discoveries and insights**. *Annu Rev Immunol* 1996, **14**:649-683.

8. Barnes BJ, Field AE, Pitha-Rowe PM: **Virus-induced heterodimer formation between IRF-5 and IRF-7 modulates assembly of the IFNA enhanceosome in vivo and transcriptional activity of IFNA genes**. *J Biol Chem* 2003, **278**(19):16630-16641.

9. Barnes BJ, Kellum MJ, Field AE, Pitha PM: **Multiple regulatory domains of IRF-5 control activation, cellular localization, and induction of chemokines that mediate recruitment of T lymphocytes**. *Mol Cell Biol* 2002, **22**(16):5721-5740.

10. Barnes BJ, Kellum MJ, Pinder KE, Frisancho JA, Pitha PM: **Interferon regulatory factor 5, a novel mediator of cell cycle arrest and cell death**. *Cancer Res* 2003, **63**(19):6424-6431.

11. Barnes BJ, Moore PA, Pitha PM: **Virus-specific activation of a novel interferon regulatory factor, IRF-5, results in the induction of distinct interferon alpha genes**. *J Biol Chem* 2001, **276**(26):23382-23390.

12. Barnes BJ, Richards J, Mancl M, Hanash S, Beretta L, Pitha PM: **Global and distinct targets of IRF-5 and IRF-7 during innate response to viral infection**. *J Biol Chem* 2004, **279**(43):45194-45207.

13. Barton GM, Medzhitov R: **Toll-like receptor signaling pathways**. *Science* 2003, **300**(5625):1524-1525.

14. Beg AA, Ruben SM, Scheinman RI, Haskill S, Rosen CA, Baldwin AS, Jr.: **I kappa B interacts with the nuclear localization sequences of the subunits of NF-kappa B: a mechanism for cytoplasmic retention**. *Genes Dev* 1992, **6**(10):1899-1913.

15. Beinke S, Ley SC: **Functions of NF-kappaB1 and NF-kappaB2 in immune cell biology**. *Biochem J* 2004, **382**(Pt 2):393-409.

16. Benetti R, Del Sal G, Monte M, Paroni G, Brancolini C, Schneider C: **The death substrate Gas2 binds m-calpain and increases susceptibility to p53-dependent apoptosis**. *Embo J* 2001, **20**(11):2702-2714.

17. Bhattacharya S, Eckner R, Grossman S, Oldread E, Arany Z, D'Andrea A, Livingston DM: **Cooperation of Stat2 and p300/CBP in signalling induced by interferon-alpha**. *Nature* 1996, **383**(6598):344-347.

18. Bluyssen HA, Muzaffar R, Vlieststra RJ, van der Made AC, Leung S, Stark GR, Kerr IM, Trapman J, Levy DE: **Combinatorial association and abundance of components of interferon-stimulated gene factor 3 dictate the selectivity of interferon responses**. *Proc Natl Acad Sci U S A* 1995, **92**(12):5645-5649.

19. Bonzon C, Bouchier-Hayes L, Pagliari LJ, Green DR, Newmeyer DD: **Caspase-2-induced apoptosis requires bid cleavage: a physiological role for bid in heat shock-induced death**. *Mol Biol Cell* 2006, **17**(5):2150-2157.

20. Bratton SB, Walker G, Srinivasula SM, Sun XM, Butterworth M, Alnemri ES, Cohen GM: **Recruitment, activation and retention of caspases-9 and -3 by Apaf-1 apoptosome and associated XIAP complexes**. *Embo J* 2001, **20**(5):998-1009.

21. Brikos C, Wait R, Begum S, O'Neill LA, Saklatvala J: **Mass spectrometric analysis of the endogenous type I interleukin-1 (IL-1) receptor signaling complex formed after IL-1 binding identifies IL-1RAcP, MyD88, and IRAK-4 as the stable components**. *Mol Cell Proteomics* 2007, **6**(9):1551-1559.

22. Buss H, Dorrie A, Schmitz ML, Hoffmann E, Resch K, Kracht M: **Constitutive and interleukin-1-inducible phosphorylation of p65 NF-{kappa}B at serine 536 is mediated by multiple protein kinases including I{kappa}B kinase (IKK)-{alpha}, IKK{beta}, IKK{epsilon}, TRAF family member-associated (TANK)-binding kinase 1 (TBK1), and an unknown kinase and couples p65 to TATA-binding protein-associated factor II31-mediated interleukin-8 transcription**. *J Biol Chem* 2004, **279**(53):55633-55643.

23. Caillaud A, Prakash A, Smith E, Masumi A, Hovanessian AG, Levy DE, Marie I: **Acetylation of interferon regulatory factor-7 by p300/CREB-binding protein (CBP)-associated factor (PCAF) impairs its DNA binding**. *J Biol Chem* 2002, **277**(51):49417-49421.

24. Chang HY, Nishitoh H, Yang X, Ichijo H, Baltimore D: **Activation of apoptosis signal-regulating kinase 1 (ASK1) by the adapter protein Daxx**. *Science* 1998, **281**(5384):1860-1863.

25. Chatterjee-Kishore M, van Den Akker F, Stark GR: **Adenovirus E1A down-regulates LMP2 transcription by interfering with the binding of stat1 to IRF1**. *J Biol Chem* 2000, **275**(27):20406-20411.

26. Chaudhary PM, Eby M, Jasmin A, Bookwalter A, Murray J, Hood L: **Death receptor 5, a new member of the TNFR family, and DR4 induce FADD-dependent apoptosis and activate the NF-kappaB pathway**. *Immunity* 1997, **7**(6):821-830.

27. Chen PC, DuBois GC, Chen MJ: **Mapping the domain(s) critical for the binding of human tumor necrosis factor-alpha to its two receptors**. *J Biol Chem* 1995, **270**(6):2874-2878.

28. Cheng H, Addona T, Keshishian H, Dahlstrand E, Lu C, Dorsch M, Li Z, Wang A, Ocain TD, Li P *et al*: **Regulation of IRAK-4 kinase activity via autophosphorylation within its activation loop**. *Biochem Biophys Res Commun* 2007, **352**(3):609-616.

29. Childs KS, Goodbourn S: **Identification of novel co-repressor molecules for Interferon Regulatory Factor-2**. *Nucleic Acids Res* 2003, **31**(12):3016-3026.

30. Chinnaiyan AM, O'Rourke K, Tewari M, Dixit VM: **FADD, a novel death domain-containing protein, interacts with the death domain of Fas and initiates apoptosis**. *Cell* 1995, **81**(4):505-512.

31. Chinnaiyan AM, O'Rourke K, Yu GL, Lyons RH, Garg M, Duan DR, Xing L, Gentz R, Ni J, Dixit VM: **Signal transduction by DR3, a death domain-containing receptor related to TNFR-1 and CD95**. *Science* 1996, **274**(5289):990-992.

32. Chow WA, Fang JJ, Yee JK: **The IFN regulatory factor family participates in regulation of Fas ligand gene expression in T cells**. *J Immunol* 2000, **164**(7):3512-3518.

33. Chu K, Niu X, Williams LT: **A Fas-associated protein factor, FAF1, potentiates Fas-mediated apoptosis**. *Proc Natl Acad Sci U S A* 1995, **92**(25):11894-11898.

34. Clarke N, Jimenez-Lara AM, Voltz E, Gronemeyer H: **Tumor suppressor IRF-1 mediates retinoid and interferon anticancer signaling to death ligand TRAIL**. *Embo J* 2004, **23**(15):3051-3060.

35. Clarke P, Debiasi RL, Meintzer SM, Robinson BA, Tyler KL: **Inhibition of NF-kappa B activity and cFLIP expression contribute to viral-induced apoptosis**. *Apoptosis* 2005, **10**(3):513-524.

36. Clemens MJ, Elia A: **The double-stranded RNA-dependent protein kinase PKR: structure and function**. *J Interferon Cytokine Res* 1997, **17**(9):503-524.

37. Cohen B, Novick D, Barak S, Rubinstein M: **Ligand-induced association of the type I interferon receptor components**. *Mol Cell Biol* 1995, **15**(8):4208-4214.

38. Colamonici O, Yan H, Domanski P, Handa R, Smalley D, Mullersman J, Witte M, Krishnan K, Krolewski J: **Direct binding to and tyrosine phosphorylation of the alpha subunit of the type I interferon receptor by p135tyk2 tyrosine kinase**. *Mol Cell Biol* 1994, **14**(12):8133-8142.

39. Cuddihy AR, Li S, Tam NW, Wong AH, Taya Y, Abraham N, Bell JC, Koromilas AE: **Double-stranded-RNA-activated protein kinase PKR enhances transcriptional activation by tumor suppressor p53**. *Mol Cell Biol* 1999, **19**(4):2475-2484.

40. Cuddihy AR, Wong AH, Tam NW, Li S, Koromilas AE: **The double-stranded RNA activated protein kinase PKR physically associates with the tumor suppressor p53 protein and phosphorylates human p53 on serine 392 in vitro**. *Oncogene* 1999, **18**(17):2690-2702.

41. Cusson-Hermance N, Khurana S, Lee TH, Fitzgerald KA, Kelliher MA: **Rip1 mediates the Trif-dependent toll-like receptor 3- and 4-induced NF-{kappa}B activation but does not contribute to interferon regulatory factor 3 activation**. *J Biol Chem* 2005, **280**(44):36560-36566.

42. Danial NN, Korsmeyer SJ: **Cell death: critical control points**. *Cell* 2004, **116**(2):205-219.

43. Das KC, Lewis-Molock Y, White CW: **Thiol modulation of TNF alpha and IL-1 induced MnSOD gene expression and activation of NF-kappa B**. *Mol Cell Biochem* 1995, **148**(1):45-57.

44. De Lucca GV, Kim UT, Liang J, Cordova B, Klabe RM, Garber S, Bacheler LT, Lam GN, Wright MR, Logue KA *et al*: **Nonsymmetric P2/P2' cyclic urea HIV protease inhibitors. Structure-activity relationship, bioavailability, and resistance profile of monoindazole-substituted P2 analogues**. *J Med Chem* 1998, **41**(13):2411-2423.

45. Dejardin E, Droin NM, Delhase M, Haas E, Cao Y, Makris C, Li ZW, Karin M, Ware CF, Green DR: **The lymphotoxin-beta receptor induces different patterns of gene expression via two NF-kappaB pathways**. *Immunity* 2002, **17**(4):525-535.

46. Delhalle S, Blasius R, Dicato M, Diederich M: **A beginner's guide to NF-kappaB signaling pathways**. *Ann N Y Acad Sci* 2004, **1030**:1-13.

47. Deng Y, Lin Y, Wu X: **TRAIL-induced apoptosis requires Bax-dependent mitochondrial release of Smac/DIABLO**. *Genes Dev* 2002, **16**(1):33-45.

48. Der SD, Zhou A, Williams BR, Silverman RH: **Identification of genes differentially regulated by interferon alpha, beta, or gamma using oligonucleotide arrays**. *Proc Natl Acad Sci U S A* 1998, **95**(26):15623-15628.

49. Deveraux QL, Roy N, Stennicke HR, Van Arsdale T, Zhou Q, Srinivasula SM, Alnemri ES, Salvesen GS, Reed JC: **IAPs block apoptotic events induced by caspase-8 and cytochrome c by direct inhibition of distinct caspases**. *Embo J* 1998, **17**(8):2215-2223.

50. Deveraux QL, Takahashi R, Salvesen GS, Reed JC: **X-linked IAP is a direct inhibitor of cell-death proteases**. *Nature* 1997, **388**(6639):300-304.

51. Devin A, Cook A, Lin Y, Rodriguez Y, Kelliher M, Liu Z: **The distinct roles of TRAF2 and RIP in IKK activation by TNF-R1: TRAF2 recruits IKK to TNF-R1 while RIP mediates IKK activation**. *Immunity* 2000, **12**(4):419-429.

52. Devin A, Lin Y, Yamaoka S, Li Z, Karin M, Liu Z: **The alpha and beta subunits of IkappaB kinase (IKK) mediate TRAF2-dependent IKK recruitment to tumor necrosis factor (TNF) receptor 1 in response to TNF**. *Mol Cell Biol* 2001, **21**(12):3986-3994.

53. Diaz JL, Oltersdorf T, Horne W, McConnell M, Wilson G, Weeks S, Garcia T, Fritz LC: **A common binding site mediates heterodimerization and homodimerization of Bcl-2 family members**. *J Biol Chem* 1997, **272**(17):11350-11355.

54. Domanski P, Fish E, Nadeau OW, Witte M, Platanias LC, Yan H, Krolewski J, Pitha P, Colamonici OR: **A region of the beta subunit of the interferon alpha receptor different from box 1 interacts with Jak1 and is sufficient to activate the Jak-Stat pathway and induce an antiviral state**. *J Biol Chem* 1997, **272**(42):26388-26393.

55. D'Souza BN, Edelstein LC, Pegman PM, Smith SM, Loughran ST, Clarke A, Mehl A, Rowe M, Gelinas C, Walls D: **Nuclear factor kappa B-dependent activation of the antiapoptotic bfl-1 gene by the Epstein-Barr virus latent membrane protein 1 and activated CD40 receptor**. *J Virol* 2004, **78**(4):1800-1816.

56. Duran A, Diaz-Meco MT, Moscat J: **Essential role of RelA Ser311 phosphorylation by zetaPKC in NF-kappaB transcriptional activation**. *Embo J* 2003, **22**(15):3910-3918.

57. Earnshaw WC, Martins LM, Kaufmann SH: **Mammalian caspases: structure, activation, substrates, and functions during apoptosis**. *Annu Rev Biochem* 1999, **68**:383-424.

58. Edelstein LC, Lagos L, Simmons M, Tirumalai H, Gelinas C: **NF-kappa B-dependent assembly of an enhanceosome-like complex on the promoter region of apoptosis inhibitor Bfl-1/A1**. *Mol Cell Biol* 2003, **23**(8):2749-2761.

59. Eklund EA, Jalava A, Kakar R: **PU.1, interferon regulatory factor 1, and interferon consensus sequence-binding protein cooperate to increase gp91(phox) expression**. *J Biol Chem* 1998, **273**(22):13957-13965.

60. Enari M, Sakahira H, Yokoyama H, Okawa K, Iwamatsu A, Nagata S: **A caspase-activated DNase that degrades DNA during apoptosis, and its inhibitor ICAD**. *Nature* 1998, **391**(6662):43-50.

61. Endo TA, Masuhara M, Yokouchi M, Suzuki R, Sakamoto H, Mitsui K, Matsumoto A, Tanimura S, Ohtsubo M, Misawa H *et al*: **A new protein containing an SH2 domain that inhibits JAK kinases**. *Nature* 1997, **387**(6636):921-924.

62. Erl W, Hansson GK, de Martin R, Draude G, Weber KS, Weber C: **Nuclear factor-kappa B regulates induction of apoptosis and inhibitor of apoptosis protein-1 expression in vascular smooth muscle cells**. *Circ Res* 1999, **84**(6):668-677.

63. Fernandes-Alnemri T, Armstrong RC, Krebs J, Srinivasula SM, Wang L, Bullrich F, Fritz LC, Trapani JA, Tomaselli KJ, Litwack G *et al*: **In vitro activation of CPP32 and Mch3 by Mch4, a novel human apoptotic cysteine protease containing two FADD-like domains**. *Proc Natl Acad Sci U S A* 1996, **93**(15):7464-7469.

64. Fitzgerald KA, Palsson-McDermott EM, Bowie AG, Jefferies CA, Mansell AS, Brady G, Brint E, Dunne A, Gray P, Harte MT *et al*: **Mal (MyD88-adapter-like) is required for Toll-like receptor-4 signal transduction**. *Nature* 2001, **413**(6851):78-83.

65. Fortin A, Cregan SP, MacLaurin JG, Kushwaha N, Hickman ES, Thompson CS, Hakim A, Albert PR, Cecconi F, Helin K *et al*: **APAF1 is a key transcriptional target for p53 in the regulation of neuronal cell death**. *J Cell Biol* 2001, **155**(2):207-216.

66. Fu XY: **A transcription factor with SH2 and SH3 domains is directly activated by an interferon alpha-induced cytoplasmic protein tyrosine kinase(s)**. *Cell* 1992, **70**(2):323-335.

67. Galabru J, Katze MG, Robert N, Hovanessian AG: **The binding of double-stranded RNA and adenovirus VAI RNA to the interferon-induced protein kinase**. *Eur J Biochem* 1989, **178**(3):581-589.

68. Gauzzi MC, Velazquez L, McKendry R, Mogensen KE, Fellous M, Pellegrini S: **Interferon-alpha-dependent activation of Tyk2 requires phosphorylation of positive regulatory tyrosines by another kinase**. *J Biol Chem* 1996, **271**(34):20494-20500.

69. Glasgow JN, Qiu J, Rassin D, Grafe M, Wood T, Perez-Pol JR: **Transcriptional regulation of the BCL-X gene by NF-kappaB is an element of hypoxic responses in the rat brain**. *Neurochem Res* 2001, **26**(6):647-659.

70. Greenlund AC, Morales MO, Viviano BL, Yan H, Krolewski J, Schreiber RD: **Stat recruitment by tyrosine-phosphorylated cytokine receptors: an ordered reversible affinity-driven process**. *Immunity* 1995, **2**(6):677-687.

71. Gross A, Yin XM, Wang K, Wei MC, Jockel J, Milliman C, Erdjument-Bromage H, Tempst P, Korsmeyer SJ: **Caspase cleaved BID targets mitochondria and is required for cytochrome c release, while BCL-XL prevents this release but not tumor necrosis factor-R1/Fas death**. *J Biol Chem* 1999, **274**(2):1156-1163.

72. Guo Y, Srinivasula SM, Druilhe A, Fernandes-Alnemri T, Alnemri ES: **Caspase-2 induces apoptosis by releasing proapoptotic proteins from mitochondria**. *J Biol Chem* 2002, **277**(16):13430-13437.

73. Hakem R, Hakem A, Duncan GS, Henderson JT, Woo M, Soengas MS, Elia A, de la Pompa JL, Kagi D, Khoo W *et al*: **Differential requirement for caspase 9 in apoptotic pathways in vivo**. *Cell* 1998, **94**(3):339-352.

74. Han J, Flemington C, Houghton AB, Gu Z, Zambetti GP, Lutz RJ, Zhu L, Chittenden T: **Expression of bbc3, a pro-apoptotic BH3-only gene, is regulated by diverse cell death and survival signals**. *Proc Natl Acad Sci U S A* 2001, **98**(20):11318-11323.

75. Han KJ, Su X, Xu LG, Bin LH, Zhang J, Shu HB: **Mechanisms of the TRIF-induced interferon-stimulated response element and NF-kappaB activation and apoptosis pathways**. *J Biol Chem* 2004, **279**(15):15652-15661.

76. Harper N, Hughes M, MacFarlane M, Cohen GM: **Fas-associated death domain protein and caspase-8 are not recruited to the tumor necrosis factor receptor 1 signaling complex during tumor necrosis factor-induced apoptosis**. *J Biol Chem* 2003, **278**(28):25534-25541.

77. Haupt Y, Maya R, Kazaz A, Oren M: **Mdm2 promotes the rapid degradation of p53**. *Nature* 1997, **387**(6630):296-299.

78. Heinrich PC, Behrmann I, Muller-Newen G, Schaper F, Graeve L: **Interleukin-6-type cytokine signalling through the gp130/Jak/STAT pathway**. *Biochem J* 1998, **334 ( Pt 2)**:297-314.

79. Hemmi S, Bohni R, Stark G, Di Marco F, Aguet M: **A novel member of the interferon receptor family complements functionality of the murine interferon gamma receptor in human cells**. *Cell* 1994, **76**(5):803-810.

80. Hemmi S, Peghini P, Metzler M, Merlin G, Dembic Z, Aguet M: **Cloning of murine interferon gamma receptor cDNA: expression in human cells mediates high-affinity binding but is not sufficient to confer sensitivity to murine interferon gamma**. *Proc Natl Acad Sci U S A* 1989, **86**(24):9901-9905.

81. Henshall DC, Bonislawski DP, Skradski SL, Araki T, Lan JQ, Schindler CK, Meller R, Simon RP: **Formation of the Apaf-1/cytochrome c complex precedes activation of caspase-9 during seizure-induced neuronal death**. *Cell Death Differ* 2001, **8**(12):1169-1181.

82. Hershey JW: **Protein phosphorylation controls translation rates**. *J Biol Chem* 1989, **264**(35):20823-20826.

83. Hibino Y, Kumar CS, Mariano TM, Lai DH, Pestka S: **Chimeric interferon-gamma receptors demonstrate that an accessory factor required for activity interacts with the extracellular domain**. *J Biol Chem* 1992, **267**(6):3741-3749.

84. Hickman ES, Helin K: **The regulation of APAF1 expression during development and tumourigenesis**. *Apoptosis* 2002, **7**(2):167-171.

85. Hirata H, Takahashi A, Kobayashi S, Yonehara S, Sawai H, Okazaki T, Yamamoto K, Sasada M: **Caspases are activated in a branched protease cascade and control distinct downstream processes in Fas-induced apoptosis**. *J Exp Med* 1998, **187**(4):587-600.

86. Hirata Y, Maeda S, Ohmae T, Shibata W, Yanai A, Ogura K, Yoshida H, Kawabe T, Omata M: **Helicobacter pylori induces IkappaB kinase alpha nuclear translocation and chemokine production in gastric epithelial cells**. *Infect Immun* 2006, **74**(3):1452-1461.

87. Honda K, Yanai H, Takaoka A, Taniguchi T: **Regulation of the type I IFN induction: a current view**. *Int Immunol* 2005, **17**(11):1367-1378.

88. Horvath CM, Stark GR, Kerr IM, Darnell JE, Jr.: **Interactions between STAT and non-STAT proteins in the interferon-stimulated gene factor 3 transcription complex**. *Mol Cell Biol* 1996, **16**(12):6957-6964.

89. Hosui A, Ohkawa K, Ishida H, Sato A, Nakanishi F, Ueda K, Takehara T, Kasahara A, Sasaki Y, Hori M *et al*: **Hepatitis C virus core protein differently regulates the JAK-STAT signaling pathway under interleukin-6 and interferon-gamma stimuli**. *J Biol Chem* 2003, **278**(31):28562-28571.

90. Hsu H, Huang J, Shu HB, Baichwal V, Goeddel DV: **TNF-dependent recruitment of the protein kinase RIP to the TNF receptor-1 signaling complex**. *Immunity* 1996, **4**(4):387-396.

91. Hsu H, Shu HB, Pan MG, Goeddel DV: **TRADD-TRAF2 and TRADD-FADD interactions define two distinct TNF receptor 1 signal transduction pathways**. *Cell* 1996, **84**(2):299-308.

92. Hsu H, Xiong J, Goeddel DV: **The TNF receptor 1-associated protein TRADD signals cell death and NF-kappa B activation**. *Cell* 1995, **81**(4):495-504.

93. Huang B, Eberstadt M, Olejniczak ET, Meadows RP, Fesik SW: **NMR structure and mutagenesis of the Fas (APO-1/CD95) death domain**. *Nature* 1996, **384**(6610):638-641.

94. Huang X, Hutchins B, Patel RC: **The C-terminal, third conserved motif of the protein activator PACT plays an essential role in the activation of double-stranded-RNA-dependent protein kinase (PKR)**. *Biochem J* 2002, **366**(Pt 1):175-186.

95. Huang YH, Wu JY, Zhang Y, Wu MX: **Synergistic and opposing regulation of the stress-responsive gene IEX-1 by p53, c-Myc, and multiple NF-kappaB/rel complexes**. *Oncogene* 2002, **21**(44):6819-6828.

96. Hymowitz SG, Christinger HW, Fuh G, Ultsch M, O'Connell M, Kelley RF, Ashkenazi A, de Vos AM: **Triggering cell death: the crystal structure of Apo2L/TRAIL in a complex with death receptor 5**. *Mol Cell* 1999, **4**(4):563-571.

97. Ishihara Y, Shimamoto N: **Involvement of endonuclease G in nucleosomal DNA fragmentation under sustained endogenous oxidative stress**. *J Biol Chem* 2006, **281**(10):6726-6733.

98. Izeradjene K, Douglas L, Delaney A, Houghton JA: **Influence of casein kinase II in tumor necrosis factor-related apoptosis-inducing ligand-induced apoptosis in human rhabdomyosarcoma cells**. *Clin Cancer Res* 2004, **10**(19):6650-6660.

99. Jiang HY, Wek RC: **Phosphorylation of the alpha-subunit of the eukaryotic initiation factor-2 (eIF2alpha) reduces protein synthesis and enhances apoptosis in response to proteasome inhibition**. *J Biol Chem* 2005, **280**(14):14189-14202.

100. Jiang Y, Woronicz JD, Liu W, Goeddel DV: **Prevention of constitutive TNF receptor 1 signaling by silencer of death domains**. *Science* 1999, **283**(5401):543-546.

101. Jin Z, El-Deiry WS: **Overview of cell death signaling pathways**. *Cancer Biol Ther* 2005, **4**(2):139-163.

102. Kalvakolanu DV: **Alternate interferon signaling pathways**. *Pharmacol Ther* 2003, **100**(1):1-29.

103. Kandasamy K, Srinivasula SM, Alnemri ES, Thompson CB, Korsmeyer SJ, Bryant JL, Srivastava RK: **Involvement of proapoptotic molecules Bax and Bak in tumor necrosis factor-related apoptosis-inducing ligand (TRAIL)-induced mitochondrial disruption and apoptosis: differential regulation of cytochrome c and Smac/DIABLO release**. *Cancer Res* 2003, **63**(7):1712-1721.

104. Kang SJ, Sanchez I, Jing N, Yuan J: **Dissociation between neurodegeneration and caspase-11-mediated activation of caspase-1 and caspase-3 in a mouse model of amyotrophic lateral sclerosis**. *J Neurosci* 2003, **23**(13):5455-5460.

105. Kaplan DH, Greenlund AC, Tanner JW, Shaw AS, Schreiber RD: **Identification of an interferon-gamma receptor alpha chain sequence required for JAK-1 binding**. *J Biol Chem* 1996, **271**(1):9-12.

106. Karpova AY, Ronco LV, Howley PM: **Functional characterization of interferon regulatory factor 3a (IRF-3a), an alternative splice isoform of IRF-3**. *Mol Cell Biol* 2001, **21**(13):4169-4176.

107. Kaufmann M, Bozic D, Briand C, Bodmer JL, Zerbe O, Kohl A, Tschopp J, Grutter MG: **Identification of a basic surface area of the FADD death effector domain critical for apoptotic signaling**. *FEBS Lett* 2002, **527**(1-3):250-254.

108. Kelekar A, Chang BS, Harlan JE, Fesik SW, Thompson CB: **Bad is a BH3 domain-containing protein that forms an inactivating dimer with Bcl-XL**. *Mol Cell Biol* 1997, **17**(12):7040-7046.

109. Khoshnan A, Tindell C, Laux I, Bae D, Bennett B, Nel AE: **The NF-kappa B cascade is important in Bcl-xL expression and for the anti-apoptotic effects of the CD28 receptor in primary human CD4+ lymphocytes**. *J Immunol* 2000, **165**(4):1743-1754.

110. Kischkel FC, Hellbardt S, Behrmann I, Germer M, Pawlita M, Krammer PH, Peter ME: **Cytotoxicity-dependent APO-1 (Fas/CD95)-associated proteins form a death-inducing signaling complex (DISC) with the receptor**. *Embo J* 1995, **14**(22):5579-5588.

111. Kondo T, Minamino N, Nagamura-Inoue T, Matsumoto M, Taniguchi T, Tanaka N: **Identification and characterization of nucleophosmin/B23/numatrin which binds the anti-oncogenic transcription factor IRF-1 and manifests oncogenic activity**. *Oncogene* 1997, **15**(11):1275-1281.

112. Lassus P, Opitz-Araya X, Lazebnik Y: **Requirement for caspase-2 in stress-induced apoptosis before mitochondrial permeabilization**. *Science* 2002, **297**(5585):1352-1354.

113. Lee SC, Chan J, Clement MV, Pervaiz S: **Functional proteomics of resveratrol-induced colon cancer cell apoptosis: caspase-6-mediated cleavage of lamin A is a major signaling loop**. *Proteomics* 2006, **6**(8):2386-2394.

114. Leitges M, Sanz L, Martin P, Duran A, Braun U, Garcia JF, Camacho F, Diaz-Meco MT, Rennert PD, Moscat J: **Targeted disruption of the zetaPKC gene results in the impairment of the NF-kappaB pathway**. *Mol Cell* 2001, **8**(4):771-780.

115. Leung S, Qureshi SA, Kerr IM, Darnell JE, Jr., Stark GR: **Role of STAT2 in the alpha interferon signaling pathway**. *Mol Cell Biol* 1995, **15**(3):1312-1317.

116. Li H, Zhu H, Xu CJ, Yuan J: **Cleavage of BID by caspase 8 mediates the mitochondrial damage in the Fas pathway of apoptosis**. *Cell* 1998, **94**(4):491-501.

117. Li LY, Luo X, Wang X: **Endonuclease G is an apoptotic DNase when released from mitochondria**. *Nature* 2001, **412**(6842):95-99.

118. Li Q, Verma IM: **NF-kappaB regulation in the immune system**. *Nat Rev Immunol* 2002, **2**(10):725-734.

119. Li S, Strelow A, Fontana EJ, Wesche H: **IRAK-4: a novel member of the IRAK family with the properties of an IRAK-kinase**. *Proc Natl Acad Sci U S A* 2002, **99**(8):5567-5572.

120. Lin MT, Chang CC, Chen ST, Chang HL, Su JL, Chau YP, Kuo ML: **Cyr61 expression confers resistance to apoptosis in breast cancer MCF-7 cells by a mechanism of NF-kappaB-dependent XIAP up-regulation**. *J Biol Chem* 2004, **279**(23):24015-24023.

121. Lin R, Hiscott J: **A role for casein kinase II phosphorylation in the regulation of IRF-1 transcriptional activity**. *Mol Cell Biochem* 1999, **191**(1-2):169-180.

122. Ling L, Cao Z, Goeddel DV: **NF-kappaB-inducing kinase activates IKK-alpha by phosphorylation of Ser-176**. *Proc Natl Acad Sci U S A* 1998, **95**(7):3792-3797.

123. Little SA, Kim WK, Mirkes PE: **Teratogen-induced activation of caspase-6 and caspase-7 in early postimplantation mouse embryos**. *Cell Biol Toxicol* 2003, **19**(4):215-226.

124. Liu J, Guan X, Ma X: **Interferon regulatory factor 1 is an essential and direct transcriptional activator for interferon {gamma}-induced RANTES/CCl5 expression in macrophages**. *J Biol Chem* 2005, **280**(26):24347-24355.

125. Lund JM, Alexopoulou L, Sato A, Karow M, Adams NC, Gale NW, Iwasaki A, Flavell RA: **Recognition of single-stranded RNA viruses by Toll-like receptor 7**. *Proc Natl Acad Sci U S A* 2004, **101**(15):5598-5603.

126. Luo X, Budihardjo I, Zou H, Slaughter C, Wang X: **Bid, a Bcl2 interacting protein, mediates cytochrome c release from mitochondria in response to activation of cell surface death receptors**. *Cell* 1998, **94**(4):481-490.

127. Lye E, Mirtsos C, Suzuki N, Suzuki S, Yeh WC: **The role of interleukin 1 receptor-associated kinase-4 (IRAK-4) kinase activity in IRAK-4-mediated signaling**. *J Biol Chem* 2004, **279**(39):40653-40658.

128. MacFarlane M: **TRAIL-induced signalling and apoptosis**. *Toxicol Lett* 2003, **139**(2-3):89-97.

129. Madesh M, Antonsson B, Srinivasula SM, Alnemri ES, Hajnoczky G: **Rapid kinetics of tBid-induced cytochrome c and Smac/DIABLO release and mitochondrial depolarization**. *J Biol Chem* 2002, **277**(7):5651-5659.

130. Mahalingam S, Chaudhri G, Tan CL, John A, Foster PS, Karupiah G: **Transcription of the interferon gamma (IFN-gamma )-inducible chemokine Mig in IFN-gamma-deficient mice**. *J Biol Chem* 2001, **276**(10):7568-7574.

131. Maianski NA, Roos D, Kuijpers TW: **Bid truncation, bid/bax targeting to the mitochondria, and caspase activation associated with neutrophil apoptosis are inhibited by granulocyte colony-stimulating factor**. *J Immunol* 2004, **172**(11):7024-7030.

132. Mamane Y, Heylbroeck C, Genin P, Algarte M, Servant MJ, LePage C, DeLuca C, Kwon H, Lin R, Hiscott J: **Interferon regulatory factors: the next generation**. *Gene* 1999, **237**(1):1-14.

133. Mancl ME, Hu G, Sangster-Guity N, Olshalsky SL, Hoops K, Fitzgerald-Bocarsly P, Pitha PM, Pinder K, Barnes BJ: **Two discrete promoters regulate the alternatively spliced human interferon regulatory factor-5 isoforms. Multiple isoforms with distinct cell type-specific expression, localization, regulation, and function**. *J Biol Chem* 2005, **280**(22):21078-21090.

134. Mansell A, Brint E, Gould JA, O'Neill LA, Hertzog PJ: **Mal interacts with tumor necrosis factor receptor-associated factor (TRAF)-6 to mediate NF-kappaB activation by toll-like receptor (TLR)-2 and TLR4**. *J Biol Chem* 2004, **279**(36):37227-37230.

135. Marecki S, Riendeau CJ, Liang MD, Fenton MJ: **PU.1 and multiple IFN regulatory factor proteins synergize to mediate transcriptional activation of the human IL-1 beta gene**. *J Immunol* 2001, **166**(11):6829-6838.

136. Martinez-Moczygemba M, Gutch MJ, French DL, Reich NC: **Distinct STAT structure promotes interaction of STAT2 with the p48 subunit of the interferon-alpha-stimulated transcription factor ISGF3**. *J Biol Chem* 1997, **272**(32):20070-20076.

137. Melnik AK: **A cephalometric study of mandibular asymmetry in a longitudinally followed sample of growing children**. *Am J Orthod Dentofacial Orthop* 1992, **101**(4):355-366.

138. Meraro D, Gleit-Kielmanowicz M, Hauser H, Levi BZ: **IFN-stimulated gene 15 is synergistically activated through interactions between the myelocyte/lymphocyte-specific transcription factors, PU.1, IFN regulatory factor-8/IFN consensus sequence binding protein, and IFN regulatory factor-4: characterization of a new subtype of IFN-stimulated response element**. *J Immunol* 2002, **168**(12):6224-6231.

139. Meylan E, Burns K, Hofmann K, Blancheteau V, Martinon F, Kelliher M, Tschopp J: **RIP1 is an essential mediator of Toll-like receptor 3-induced NF-kappa B activation**. *Nat Immunol* 2004, **5**(5):503-507.

140. Micheau O, Tschopp J: **Induction of TNF receptor I-mediated apoptosis via two sequential signaling complexes**. *Cell* 2003, **114**(2):181-190.

141. Miyazaki T, Reed JC: **A GTP-binding adapter protein couples TRAIL receptors to apoptosis-inducing proteins**. *Nat Immunol* 2001, **2**(6):493-500.

142. Mizel SB, Snipes JA: **Gram-negative flagellin-induced self-tolerance is associated with a block in interleukin-1 receptor-associated kinase release from toll-like receptor 5**. *J Biol Chem* 2002, **277**(25):22414-22420.

143. Muller M, Briscoe J, Laxton C, Guschin D, Ziemiecki A, Silvennoinen O, Harpur AG, Barbieri G, Witthuhn BA, Schindler C *et al*: **The protein tyrosine kinase JAK1 complements defects in interferon-alpha/beta and -gamma signal transduction**. *Nature* 1993, **366**(6451):129-135.

144. Nagata S: **Apoptosis by death factor**. *Cell* 1997, **88**(3):355-365.

145. Nair JS, DaFonseca CJ, Tjernberg A, Sun W, Darnell JE, Jr., Chait BT, Zhang JJ: **Requirement of Ca2+ and CaMKII for Stat1 Ser-727 phosphorylation in response to IFN-gamma**. *Proc Natl Acad Sci U S A* 2002, **99**(9):5971-5976.

146. Nakao Y, Funami K, Kikkawa S, Taniguchi M, Nishiguchi M, Fukumori Y, Seya T, Matsumoto M: **Surface-expressed TLR6 participates in the recognition of diacylated lipopeptide and peptidoglycan in human cells**. *J Immunol* 2005, **174**(3):1566-1573.

147. Narita M, Shimizu S, Ito T, Chittenden T, Lutz RJ, Matsuda H, Tsujimoto Y: **Bax interacts with the permeability transition pore to induce permeability transition and cytochrome c release in isolated mitochondria**. *Proc Natl Acad Sci U S A* 1998, **95**(25):14681-14686.

148. Negishi H, Ohba Y, Yanai H, Takaoka A, Honma K, Yui K, Matsuyama T, Taniguchi T, Honda K: **Negative regulation of Toll-like-receptor signaling by IRF-4**. *Proc Natl Acad Sci U S A* 2005, **102**(44):15989-15994.

149. Nguyen H, Hiscott J, Pitha PM: **The growing family of interferon regulatory factors**. *Cytokine Growth Factor Rev* 1997, **8**(4):293-312.

150. Nguyen H, Lin R, Hiscott J: **Activation of multiple growth regulatory genes following inducible expression of IRF-1 or IRF/RelA fusion proteins**. *Oncogene* 1997, **15**(12):1425-1435.

151. Nicholson DW, Ali A, Thornberry NA, Vaillancourt JP, Ding CK, Gallant M, Gareau Y, Griffin PR, Labelle M, Lazebnik YA *et al*: **Identification and inhibition of the ICE/CED-3 protease necessary for mammalian apoptosis**. *Nature* 1995, **376**(6535):37-43.

152. Nutt LK, Pataer A, Pahler J, Fang B, Roth J, McConkey DJ, Swisher SG: **Bax and Bak promote apoptosis by modulating endoplasmic reticular and mitochondrial Ca2+ stores**. *J Biol Chem* 2002, **277**(11):9219-9225.

153. Ogasawara K, Hida S, Azimi N, Tagaya Y, Sato T, Yokochi-Fukuda T, Waldmann TA, Taniguchi T, Taki S: **Requirement for IRF-1 in the microenvironment supporting development of natural killer cells**. *Nature* 1998, **391**(6668):700-703.

154. Oltvai ZN, Milliman CL, Korsmeyer SJ: **Bcl-2 heterodimerizes in vivo with a conserved homolog, Bax, that accelerates programmed cell death**. *Cell* 1993, **74**(4):609-619.

155. Oshiumi H, Matsumoto M, Funami K, Akazawa T, Seya T: **TICAM-1, an adaptor molecule that participates in Toll-like receptor 3-mediated interferon-beta induction**. *Nat Immunol* 2003, **4**(2):161-167.

156. Oshiumi H, Sasai M, Shida K, Fujita T, Matsumoto M, Seya T: **TIR-containing adapter molecule (TICAM)-2, a bridging adapter recruiting to toll-like receptor 4 TICAM-1 that induces interferon-beta**. *J Biol Chem* 2003, **278**(50):49751-49762.

157. Ottilie S, Diaz JL, Horne W, Chang J, Wang Y, Wilson G, Chang S, Weeks S, Fritz LC, Oltersdorf T: **Dimerization properties of human BAD. Identification of a BH-3 domain and analysis of its binding to mutant BCL-2 and BCL-XL proteins**. *J Biol Chem* 1997, **272**(49):30866-30872.

158. Ouaaz F, Li M, Beg AA: **A critical role for the RelA subunit of nuclear factor kappaB in regulation of multiple immune-response genes and in Fas-induced cell death**. *J Exp Med* 1999, **189**(6):999-1004.

159. Owen-Schaub LB, Zhang W, Cusack JC, Angelo LS, Santee SM, Fujiwara T, Roth JA, Deisseroth AB, Zhang WW, Kruzel E *et al*: **Wild-type human p53 and a temperature-sensitive mutant induce Fas/APO-1 expression**. *Mol Cell Biol* 1995, **15**(6):3032-3040.

160. Pan G, Humke EW, Dixit VM: **Activation of caspases triggered by cytochrome c in vitro**. *FEBS Lett* 1998, **426**(1):151-154.

161. Pan G, Ni J, Wei YF, Yu G, Gentz R, Dixit VM: **An antagonist decoy receptor and a death domain-containing receptor for TRAIL**. *Science* 1997, **277**(5327):815-818.

162. Pan G, O'Rourke K, Chinnaiyan AM, Gentz R, Ebner R, Ni J, Dixit VM: **The receptor for the cytotoxic ligand TRAIL**. *Science* 1997, **276**(5309):111-113.

163. Park SY, Seol JW, Lee YJ, Cho JH, Kang HS, Kim IS, Park SH, Kim TH, Yim JH, Kim M *et al*: **IFN-gamma enhances TRAIL-induced apoptosis through IRF-1**. *Eur J Biochem* 2004, **271**(21):4222-4228.

164. Park YC, Ye H, Hsia C, Segal D, Rich RL, Liou HC, Myszka DG, Wu H: **A novel mechanism of TRAF signaling revealed by structural and functional analyses of the TRADD-TRAF2 interaction**. *Cell* 2000, **101**(7):777-787.

165. Patel CV, Handy I, Goldsmith T, Patel RC: **PACT, a stress-modulated cellular activator of interferon-induced double-stranded RNA-activated protein kinase, PKR**. *J Biol Chem* 2000, **275**(48):37993-37998.

166. Patel RC, Sen GC: **PACT, a protein activator of the interferon-induced protein kinase, PKR**. *Embo J* 1998, **17**(15):4379-4390.

167. Perkins CL, Fang G, Kim CN, Bhalla KN: **The role of Apaf-1, caspase-9, and bid proteins in etoposide- or paclitaxel-induced mitochondrial events during apoptosis**. *Cancer Res* 2000, **60**(6):1645-1653.

168. Pestka S: **The interferon receptors**. *Semin Oncol* 1997, **24**(3 Suppl 9):S9-18-S19-40.

169. Peter ME, Scaffidi C, Medema JP, Kischkel F, Krammer PH: **The death receptors**. *Results Probl Cell Differ* 1999, **23**:25-63.

170. Porta C, Hadj-Slimane R, Nejmeddine M, Pampin M, Tovey MG, Espert L, Alvarez S, Chelbi-Alix MK: **Interferons alpha and gamma induce p53-dependent and p53-independent apoptosis, respectively**. *Oncogene* 2005, **24**(4):605-615.

171. Rao P, Hsu KC, Chao MV: **Upregulation of NF-kappa B-dependent gene expression mediated by the p75 tumor necrosis factor receptor**. *J Interferon Cytokine Res* 1995, **15**(2):171-177.

172. Ravagnan L, Gurbuxani S, Susin SA, Maisse C, Daugas E, Zamzami N, Mak T, Jaattela M, Penninger JM, Garrido C *et al*: **Heat-shock protein 70 antagonizes apoptosis-inducing factor**. *Nat Cell Biol* 2001, **3**(9):839-843.

173. Regnier CH, Song HY, Gao X, Goeddel DV, Cao Z, Rothe M: **Identification and characterization of an IkappaB kinase**. *Cell* 1997, **90**(2):373-383.

174. Rhee SH, Kim H, Moyer MP, Pothoulakis C: **Role of MyD88 in phosphatidylinositol 3-kinase activation by flagellin/toll-like receptor 5 engagement in colonic epithelial cells**. *J Biol Chem* 2006, **281**(27):18560-18568.

175. Rosse T, Olivier R, Monney L, Rager M, Conus S, Fellay I, Jansen B, Borner C: **Bcl-2 prolongs cell survival after Bax-induced release of cytochrome c**. *Nature* 1998, **391**(6666):496-499.

176. Rothe M, Wong SC, Henzel WJ, Goeddel DV: **A novel family of putative signal transducers associated with the cytoplasmic domain of the 75 kDa tumor necrosis factor receptor**. *Cell* 1994, **78**(4):681-692.

177. Roy N, Deveraux QL, Takahashi R, Salvesen GS, Reed JC: **The c-IAP-1 and c-IAP-2 proteins are direct inhibitors of specific caspases**. *Embo J* 1997, **16**(23):6914-6925.

178. Ruchalski K, Mao H, Singh SK, Wang Y, Mosser DD, Li F, Schwartz JH, Borkan SC: **HSP72 inhibits apoptosis-inducing factor release in ATP-depleted renal epithelial cells**. *Am J Physiol Cell Physiol* 2003, **285**(6):C1483-1493.

179. Ruchaud S, Korfali N, Villa P, Kottke TJ, Dingwall C, Kaufmann SH, Earnshaw WC: **Caspase-6 gene disruption reveals a requirement for lamin A cleavage in apoptotic chromatin condensation**. *Embo J* 2002, **21**(8):1967-1977.

180. Ryu SW, Lee SJ, Park MY, Jun JI, Jung YK, Kim E: **Fas-associated factor 1, FAF1, is a member of Fas death-inducing signaling complex**. *J Biol Chem* 2003, **278**(26):24003-24010.

181. Sakatsume M, Igarashi K, Winestock KD, Garotta G, Larner AC, Finbloom DS: **The Jak kinases differentially associate with the alpha and beta (accessory factor) chains of the interferon gamma receptor to form a functional receptor unit capable of activating STAT transcription factors**. *J Biol Chem* 1995, **270**(29):17528-17534.

182. Sakurai H, Miyoshi H, Mizukami J, Sugita T: **Phosphorylation-dependent activation of TAK1 mitogen-activated protein kinase kinase kinase by TAB1**. *FEBS Lett* 2000, **474**(2-3):141-145.

183. Sakurai H, Miyoshi H, Toriumi W, Sugita T: **Functional interactions of transforming growth factor beta-activated kinase 1 with IkappaB kinases to stimulate NF-kappaB activation**. *J Biol Chem* 1999, **274**(15):10641-10648.

184. Sakurai H, Nishi A, Sato N, Mizukami J, Miyoshi H, Sugita T: **TAK1-TAB1 fusion protein: a novel constitutively active mitogen-activated protein kinase kinase kinase that stimulates AP-1 and NF-kappaB signaling pathways**. *Biochem Biophys Res Commun* 2002, **297**(5):1277-1281.

185. Sakurai H, Suzuki S, Kawasaki N, Nakano H, Okazaki T, Chino A, Doi T, Saiki I: **Tumor necrosis factor-alpha-induced IKK phosphorylation of NF-kappaB p65 on serine 536 is mediated through the TRAF2, TRAF5, and TAK1 signaling pathway**. *J Biol Chem* 2003, **278**(38):36916-36923.

186. Samuel CE: **The eIF-2 alpha protein kinases, regulators of translation in eukaryotes from yeasts to humans**. *J Biol Chem* 1993, **268**(11):7603-7606.

187. Sarkar SN, Peters KL, Elco CP, Sakamoto S, Pal S, Sen GC: **Novel roles of TLR3 tyrosine phosphorylation and PI3 kinase in double-stranded RNA signaling**. *Nat Struct Mol Biol* 2004, **11**(11):1060-1067.

188. Sato M, Hata N, Asagiri M, Nakaya T, Taniguchi T, Tanaka N: **Positive feedback regulation of type I IFN genes by the IFN-inducible transcription factor IRF-7**. *FEBS Lett* 1998, **441**(1):106-110.

189. Sato S, Sugiyama M, Yamamoto M, Watanabe Y, Kawai T, Takeda K, Akira S: **Toll/IL-1 receptor domain-containing adaptor inducing IFN-beta (TRIF) associates with TNF receptor-associated factor 6 and TANK-binding kinase 1, and activates two distinct transcription factors, NF-kappa B and IFN-regulatory factor-3, in the Toll-like receptor signaling**. *J Immunol* 2003, **171**(8):4304-4310.

190. Scaffidi C, Fulda S, Srinivasan A, Friesen C, Li F, Tomaselli KJ, Debatin KM, Krammer PH, Peter ME: **Two CD95 (APO-1/Fas) signaling pathways**. *Embo J* 1998, **17**(6):1675-1687.

191. Schall TJ, Lewis M, Koller KJ, Lee A, Rice GC, Wong GH, Gatanaga T, Granger GA, Lentz R, Raab H *et al*: **Molecular cloning and expression of a receptor for human tumor necrosis factor**. *Cell* 1990, **61**(2):361-370.

192. Schindler C, Fu XY, Improta T, Aebersold R, Darnell JE, Jr.: **Proteins of transcription factor ISGF-3: one gene encodes the 91-and 84-kDa ISGF-3 proteins that are activated by interferon alpha**. *Proc Natl Acad Sci U S A* 1992, **89**(16):7836-7839.

193. Schneider P, Bodmer JL, Holler N, Mattmann C, Scuderi P, Terskikh A, Peitsch MC, Tschopp J: **Characterization of Fas (Apo-1, CD95)-Fas ligand interaction**. *J Biol Chem* 1997, **272**(30):18827-18833.

194. Schneider P, Thome M, Burns K, Bodmer JL, Hofmann K, Kataoka T, Holler N, Tschopp J: **TRAIL receptors 1 (DR4) and 2 (DR5) signal FADD-dependent apoptosis and activate NF-kappaB**. *Immunity* 1997, **7**(6):831-836.

195. Schram BR, Rothstein TL: **NF-kappa B is required for surface Ig-induced Fas resistance in B cells**. *J Immunol* 2003, **170**(6):3118-3124.

196. Schroder K, Hertzog PJ, Ravasi T, Hume DA: **Interferon-gamma: an overview of signals, mechanisms and functions**. *J Leukoc Biol* 2004, **75**(2):163-189.

197. Schuler M, Green DR: **Mechanisms of p53-dependent apoptosis**. *Biochem Soc Trans* 2001, **29**(Pt 6):684-688.

198. Senftleben U, Cao Y, Xiao G, Greten FR, Krahn G, Bonizzi G, Chen Y, Hu Y, Fong A, Sun SC *et al*: **Activation by IKKalpha of a second, evolutionary conserved, NF-kappa B signaling pathway**. *Science* 2001, **293**(5534):1495-1499.

199. Senn JJ: **Toll-like receptor-2 is essential for the development of palmitate-induced insulin resistance in myotubes**. *J Biol Chem* 2006, **281**(37):26865-26875.

200. Sgorbissa A, Benetti R, Marzinotto S, Schneider C, Brancolini C: **Caspase-3 and caspase-7 but not caspase-6 cleave Gas2 in vitro: implications for microfilament reorganization during apoptosis**. *J Cell Sci* 1999, **112 ( Pt 23)**:4475-4482.

201. Sheikh MS, Burns TF, Huang Y, Wu GS, Amundson S, Brooks KS, Fornace AJ, Jr., el-Deiry WS: **p53-dependent and -independent regulation of the death receptor KILLER/DR5 gene expression in response to genotoxic stress and tumor necrosis factor alpha**. *Cancer Res* 1998, **58**(8):1593-1598.

202. Shin S, Sung BJ, Cho YS, Kim HJ, Ha NC, Hwang JI, Chung CW, Jung YK, Oh BH: **An anti-apoptotic protein human survivin is a direct inhibitor of caspase-3 and -7**. *Biochemistry* 2001, **40**(4):1117-1123.

203. Shuai K, Horvath CM, Huang LH, Qureshi SA, Cowburn D, Darnell JE, Jr.: **Interferon activation of the transcription factor Stat91 involves dimerization through SH2-phosphotyrosyl peptide interactions**. *Cell* 1994, **76**(5):821-828.

204. Shuai K, Stark GR, Kerr IM, Darnell JE, Jr.: **A single phosphotyrosine residue of Stat91 required for gene activation by interferon-gamma**. *Science* 1993, **261**(5129):1744-1746.

205. Shuai K, Ziemiecki A, Wilks AF, Harpur AG, Sadowski HB, Gilman MZ, Darnell JE: **Polypeptide signalling to the nucleus through tyrosine phosphorylation of Jak and Stat proteins**. *Nature* 1993, **366**(6455):580-583.

206. Simoncic PD, Lee-Loy A, Barber DL, Tremblay ML, McGlade CJ: **The T cell protein tyrosine phosphatase is a negative regulator of janus family kinases 1 and 3**. *Curr Biol* 2002, **12**(6):446-453.

207. Slee EA, Harte MT, Kluck RM, Wolf BB, Casiano CA, Newmeyer DD, Wang HG, Reed JC, Nicholson DW, Alnemri ES *et al*: **Ordering the cytochrome c-initiated caspase cascade: hierarchical activation of caspases-2, -3, -6, -7, -8, and -10 in a caspase-9-dependent manner**. *J Cell Biol* 1999, **144**(2):281-292.

208. Soengas MS, Alarcon RM, Yoshida H, Giaccia AJ, Hakem R, Mak TW, Lowe SW: **Apaf-1 and caspase-9 in p53-dependent apoptosis and tumor inhibition**. *Science* 1999, **284**(5411):156-159.

209. Soh J, Donnelly RJ, Kotenko S, Mariano TM, Cook JR, Wang N, Emanuel S, Schwartz B, Miki T, Pestka S: **Identification and sequence of an accessory factor required for activation of the human interferon gamma receptor**. *Cell* 1994, **76**(5):793-802.

210. Song HY, Regnier CH, Kirschning CJ, Goeddel DV, Rothe M: **Tumor necrosis factor (TNF)-mediated kinase cascades: bifurcation of nuclear factor-kappaB and c-jun N-terminal kinase (JNK/SAPK) pathways at TNF receptor-associated factor 2**. *Proc Natl Acad Sci U S A* 1997, **94**(18):9792-9796.

211. Srinivasula SM, Ahmad M, Fernandes-Alnemri T, Litwack G, Alnemri ES: **Molecular ordering of the Fas-apoptotic pathway: the Fas/APO-1 protease Mch5 is a CrmA-inhibitable protease that activates multiple Ced-3/ICE-like cysteine proteases**. *Proc Natl Acad Sci U S A* 1996, **93**(25):14486-14491.

212. Srinivasula SM, Gupta S, Datta P, Zhang Z, Hegde R, Cheong N, Fernandes-Alnemri T, Alnemri ES: **Inhibitor of apoptosis proteins are substrates for the mitochondrial serine protease Omi/HtrA2**. *J Biol Chem* 2003, **278**(34):31469-31472.

213. Starling GC, Bajorath J, Emswiler J, Ledbetter JA, Aruffo A, Kiener PA: **Identification of amino acid residues important for ligand binding to Fas**. *J Exp Med* 1997, **185**(8):1487-1492.

214. Stehlik C, de Martin R, Kumabashiri I, Schmid JA, Binder BR, Lipp J: **Nuclear factor (NF)-kappaB-regulated X-chromosome-linked iap gene expression protects endothelial cells from tumor necrosis factor alpha-induced apoptosis**. *J Exp Med* 1998, **188**(1):211-216.

215. Stennicke HR, Jurgensmeier JM, Shin H, Deveraux Q, Wolf BB, Yang X, Zhou Q, Ellerby HM, Ellerby LM, Bredesen D *et al*: **Pro-caspase-3 is a major physiologic target of caspase-8**. *J Biol Chem* 1998, **273**(42):27084-27090.

216. Stennicke HR, Salvesen GS: **Caspases - controlling intracellular signals by protease zymogen activation**. *Biochim Biophys Acta* 2000, **1477**(1-2):299-306.

217. Sun X, Yin J, Starovasnik MA, Fairbrother WJ, Dixit VM: **Identification of a novel homotypic interaction motif required for the phosphorylation of receptor-interacting protein (RIP) by RIP3**. *J Biol Chem* 2002, **277**(11):9505-9511.

218. Sun XM, Bratton SB, Butterworth M, MacFarlane M, Cohen GM: **Bcl-2 and Bcl-xL inhibit CD95-mediated apoptosis by preventing mitochondrial release of Smac/DIABLO and subsequent inactivation of X-linked inhibitor-of-apoptosis protein**. *J Biol Chem* 2002, **277**(13):11345-11351.

219. Susin SA, Lorenzo HK, Zamzami N, Marzo I, Snow BE, Brothers GM, Mangion J, Jacotot E, Costantini P, Loeffler M *et al*: **Molecular characterization of mitochondrial apoptosis-inducing factor**. *Nature* 1999, **397**(6718):441-446.

220. Suzuki Y, Imai Y, Nakayama H, Takahashi K, Takio K, Takahashi R: **A serine protease, HtrA2, is released from the mitochondria and interacts with XIAP, inducing cell death**. *Mol Cell* 2001, **8**(3):613-621.

221. Suzuki Y, Nakabayashi Y, Takahashi R: **Ubiquitin-protein ligase activity of X-linked inhibitor of apoptosis protein promotes proteasomal degradation of caspase-3 and enhances its anti-apoptotic effect in Fas-induced cell death**. *Proc Natl Acad Sci U S A* 2001, **98**(15):8662-8667.

222. Takaoka A, Yanai H, Kondo S, Duncan G, Negishi H, Mizutani T, Kano S, Honda K, Ohba Y, Mak TW *et al*: **Integral role of IRF-5 in the gene induction programme activated by Toll-like receptors**. *Nature* 2005, **434**(7030):243-249.

223. Takeda K, Takeuchi O, Akira S: **Recognition of lipopeptides by Toll-like receptors**. *J Endotoxin Res* 2002, **8**(6):459-463.

224. Takeuchi M, Rothe M, Goeddel DV: **Anatomy of TRAF2. Distinct domains for nuclear factor-kappaB activation and association with tumor necrosis factor signaling proteins**. *J Biol Chem* 1996, **271**(33):19935-19942.

225. Takeuchi O, Sato S, Horiuchi T, Hoshino K, Takeda K, Dong Z, Modlin RL, Akira S: **Cutting edge: role of Toll-like receptor 1 in mediating immune response to microbial lipoproteins**. *J Immunol* 2002, **169**(1):10-14.

226. Tang D, Kidd VJ: **Cleavage of DFF-45/ICAD by multiple caspases is essential for its function during apoptosis**. *J Biol Chem* 1998, **273**(44):28549-28552.

227. Taniguchi T, Ogasawara K, Takaoka A, Tanaka N: **IRF family of transcription factors as regulators of host defense**. *Annu Rev Immunol* 2001, **19**:623-655.

228. Taniguchi T, Takaoka A: **The interferon-alpha/beta system in antiviral responses: a multimodal machinery of gene regulation by the IRF family of transcription factors**. *Curr Opin Immunol* 2002, **14**(1):111-116.

229. ten Hoeve J, de Jesus Ibarra-Sanchez M, Fu Y, Zhu W, Tremblay M, David M, Shuai K: **Identification of a nuclear Stat1 protein tyrosine phosphatase**. *Mol Cell Biol* 2002, **22**(16):5662-5668.

230. Tewari M, Quan LT, O'Rourke K, Desnoyers S, Zeng Z, Beidler DR, Poirier GG, Salvesen GS, Dixit VM: **Yama/CPP32 beta, a mammalian homolog of CED-3, is a CrmA-inhibitable protease that cleaves the death substrate poly(ADP-ribose) polymerase**. *Cell* 1995, **81**(5):801-809.

231. Tews DS: **Characterization of initiator and effector caspase expressions in dystrophinopathies**. *Neuropathology* 2006, **26**(1):24-31.

232. Thomas LR, Stillman DJ, Thorburn A: **Regulation of Fas-associated death domain interactions by the death effector domain identified by a modified reverse two-hybrid screen**. *J Biol Chem* 2002, **277**(37):34343-34348.

233. Tschopp J, Martinon F, Hofmann K: **Apoptosis: Silencing the death receptors**. *Curr Biol* 1999, **9**(10):R381-384.

234. Turpin P, Hay RT, Dargemont C: **Characterization of IkappaBalpha nuclear import pathway**. *J Biol Chem* 1999, **274**(10):6804-6812.

235. Twiddy D, Cohen GM, Macfarlane M, Cain K: **Caspase-7 is directly activated by the approximately 700-kDa apoptosome complex and is released as a stable XIAP-caspase-7 approximately 200-kDa complex**. *J Biol Chem* 2006, **281**(7):3876-3888.

236. Uren RT, Dewson G, Bonzon C, Lithgow T, Newmeyer DD, Kluck RM: **Mitochondrial release of pro-apoptotic proteins: electrostatic interactions can hold cytochrome c but not Smac/DIABLO to mitochondrial membranes**. *J Biol Chem* 2005, **280**(3):2266-2274.

237. Vacca A, Felli MP, Palermo R, Di Mario G, Calce A, Di Giovine M, Frati L, Gulino A, Screpanti I: **Notch3 and pre-TCR interaction unveils distinct NF-kappaB pathways in T-cell development and leukemia**. *Embo J* 2006, **25**(5):1000-1008.

238. Van Loo G, Demol H, van Gurp M, Hoorelbeke B, Schotte P, Beyaert R, Zhivotovsky B, Gevaert K, Declercq W, Vandekerckhove J *et al*: **A matrix-assisted laser desorption ionization post-source decay (MALDI-PSD) analysis of proteins released from isolated liver mitochondria treated with recombinant truncated Bid**. *Cell Death Differ* 2002, **9**(3):301-308.

239. van Loo G, Schotte P, van Gurp M, Demol H, Hoorelbeke B, Gevaert K, Rodriguez I, Ruiz-Carrillo A, Vandekerckhove J, Declercq W *et al*: **Endonuclease G: a mitochondrial protein released in apoptosis and involved in caspase-independent DNA degradation**. *Cell Death Differ* 2001, **8**(12):1136-1142.

240. Verhagen AM, Ekert PG, Pakusch M, Silke J, Connolly LM, Reid GE, Moritz RL, Simpson RJ, Vaux DL: **Identification of DIABLO, a mammalian protein that promotes apoptosis by binding to and antagonizing IAP proteins**. *Cell* 2000, **102**(1):43-53.

241. Vermeulen L, De Wilde G, Van Damme P, Vanden Berghe W, Haegeman G: **Transcriptional activation of the NF-kappaB p65 subunit by mitogen- and stress-activated protein kinase-1 (MSK1)**. *Embo J* 2003, **22**(6):1313-1324.

242. Vigers GP, Anderson LJ, Caffes P, Brandhuber BJ: **Crystal structure of the type-I interleukin-1 receptor complexed with interleukin-1beta**. *Nature* 1997, **386**(6621):190-194.

243. Vincenz C, Dixit VM: **Fas-associated death domain protein interleukin-1beta-converting enzyme 2 (FLICE2), an ICE/Ced-3 homologue, is proximally involved in CD95- and p55-mediated death signaling**. *J Biol Chem* 1997, **272**(10):6578-6583.

244. Walczak H, Degli-Esposti MA, Johnson RS, Smolak PJ, Waugh JY, Boiani N, Timour MS, Gerhart MJ, Schooley KA, Smith CA *et al*: **TRAIL-R2: a novel apoptosis-mediating receptor for TRAIL**. *Embo J* 1997, **16**(17):5386-5397.

245. Walter MR, Windsor WT, Nagabhushan TL, Lundell DJ, Lunn CA, Zauodny PJ, Narula SK: **Crystal structure of a complex between interferon-gamma and its soluble high-affinity receptor**. *Nature* 1995, **376**(6537):230-235.

246. Wan XS, Devalaraja MN, St Clair DK: **Molecular structure and organization of the human manganese superoxide dismutase gene**. *DNA Cell Biol* 1994, **13**(11):1127-1136.

247. Wang CY, Mayo MW, Korneluk RG, Goeddel DV, Baldwin AS, Jr.: **NF-kappaB antiapoptosis: induction of TRAF1 and TRAF2 and c-IAP1 and c-IAP2 to suppress caspase-8 activation**. *Science* 1998, **281**(5383):1680-1683.

248. Wang GQ, Gastman BR, Wieckowski E, Goldstein LA, Gambotto A, Kim TH, Fang B, Rabinovitz A, Yin XM, Rabinowich H: **A role for mitochondrial Bak in apoptotic response to anticancer drugs**. *J Biol Chem* 2001, **276**(36):34307-34317.

249. Wang GQ, Gastman BR, Wieckowski E, Goldstein LA, Rabinovitz A, Yin XM, Rabinowich H: **Apoptosis-resistant mitochondria in T cells selected for resistance to Fas signaling**. *J Biol Chem* 2001, **276**(5):3610-3619.

250. Wang J, Chun HJ, Wong W, Spencer DM, Lenardo MJ: **Caspase-10 is an initiator caspase in death receptor signaling**. *Proc Natl Acad Sci U S A* 2001, **98**(24):13884-13888.

251. Wang Q, Wang X, Evers BM: **Induction of cIAP-2 in human colon cancer cells through PKC delta/NF-kappa B**. *J Biol Chem* 2003, **278**(51):51091-51099.

252. Wang S, Miura M, Jung YK, Zhu H, Li E, Yuan J: **Murine caspase-11, an ICE-interacting protease, is essential for the activation of ICE**. *Cell* 1998, **92**(4):501-509.

253. Watling D, Guschin D, Muller M, Silvennoinen O, Witthuhn BA, Quelle FW, Rogers NC, Schindler C, Stark GR, Ihle JN *et al*: **Complementation by the protein tyrosine kinase JAK2 of a mutant cell line defective in the interferon-gamma signal transduction pathway**. *Nature* 1993, **366**(6451):166-170.

254. Wesche H, Henzel WJ, Shillinglaw W, Li S, Cao Z: **MyD88: an adapter that recruits IRAK to the IL-1 receptor complex**. *Immunity* 1997, **7**(6):837-847.

255. Woronicz JD, Gao X, Cao Z, Rothe M, Goeddel DV: **IkappaB kinase-beta: NF-kappaB activation and complex formation with IkappaB kinase-alpha and NIK**. *Science* 1997, **278**(5339):866-869.

256. Wu GS, Burns TF, McDonald ER, 3rd, Jiang W, Meng R, Krantz ID, Kao G, Gan DD, Zhou JY, Muschel R *et al*: **KILLER/DR5 is a DNA damage-inducible p53-regulated death receptor gene**. *Nat Genet* 1997, **17**(2):141-143.

257. Wu S, Kaufman RJ: **Double-stranded (ds) RNA binding and not dimerization correlates with the activation of the dsRNA-dependent protein kinase (PKR)**. *J Biol Chem* 1996, **271**(3):1756-1763.

258. Wu S, Kaufman RJ: **A model for the double-stranded RNA (dsRNA)-dependent dimerization and activation of the dsRNA-activated protein kinase PKR**. *J Biol Chem* 1997, **272**(2):1291-1296.

259. Wu X, Bayle JH, Olson D, Levine AJ: **The p53-mdm-2 autoregulatory feedback loop**. *Genes Dev* 1993, **7**(7A):1126-1132.

260. Xi H, Eason DD, Ghosh D, Dovhey S, Wright KL, Blanck G: **Co-occupancy of the interferon regulatory element of the class II transactivator (CIITA) type IV promoter by interferon regulatory factors 1 and 2**. *Oncogene* 1999, **18**(43):5889-5903.

261. Xi H, Goodwin B, Shepherd AT, Blanck G: **Impaired class II transactivator expression in mice lacking interferon regulatory factor-2**. *Oncogene* 2001, **20**(31):4219-4227.

262. Xiao G, Harhaj EW, Sun SC: **NF-kappaB-inducing kinase regulates the processing of NF-kappaB2 p100**. *Mol Cell* 2001, **7**(2):401-409.

263. Yamaguchi H, Bhalla K, Wang HG: **Bax plays a pivotal role in thapsigargin-induced apoptosis of human colon cancer HCT116 cells by controlling Smac/Diablo and Omi/HtrA2 release from mitochondria**. *Cancer Res* 2003, **63**(7):1483-1489.

264. Yamashita K, Takahashi A, Kobayashi S, Hirata H, Mesner PW, Jr., Kaufmann SH, Yonehara S, Yamamoto K, Uchiyama T, Sasada M: **Caspases mediate tumor necrosis factor-alpha-induced neutrophil apoptosis and downregulation of reactive oxygen production**. *Blood* 1999, **93**(2):674-685.

265. Yan H, Krishnan K, Greenlund AC, Gupta S, Lim JT, Schreiber RD, Schindler CW, Krolewski JJ: **Phosphorylated interferon-alpha receptor 1 subunit (IFNaR1) acts as a docking site for the latent form of the 113 kDa STAT2 protein**. *Embo J* 1996, **15**(5):1064-1074.

266. Yan H, Krishnan K, Lim JT, Contillo LG, Krolewski JJ: **Molecular characterization of an alpha interferon receptor 1 subunit (IFNaR1) domain required for TYK2 binding and signal transduction**. *Mol Cell Biol* 1996, **16**(5):2074-2082.

267. Yang E, Zha J, Jockel J, Boise LH, Thompson CB, Korsmeyer SJ: **Bad, a heterodimeric partner for Bcl-XL and Bcl-2, displaces Bax and promotes cell death**. *Cell* 1995, **80**(2):285-291.

268. Yang RB, Mark MR, Gurney AL, Godowski PJ: **Signaling events induced by lipopolysaccharide-activated toll-like receptor 2**. *J Immunol* 1999, **163**(2):639-643.

269. Yang X, Khosravi-Far R, Chang HY, Baltimore D: **Daxx, a novel Fas-binding protein that activates JNK and apoptosis**. *Cell* 1997, **89**(7):1067-1076.

270. Ye H, Cande C, Stephanou NC, Jiang S, Gurbuxani S, Larochette N, Daugas E, Garrido C, Kroemer G, Wu H: **DNA binding is required for the apoptogenic action of apoptosis inducing factor**. *Nat Struct Biol* 2002, **9**(9):680-684.

271. Yeung MC, Lau AS: **Tumor suppressor p53 as a component of the tumor necrosis factor-induced, protein kinase PKR-mediated apoptotic pathway in human promonocytic U937 cells**. *J Biol Chem* 1998, **273**(39):25198-25202.

272. Yu J, Zhang L, Hwang PM, Kinzler KW, Vogelstein B: **PUMA induces the rapid apoptosis of colorectal cancer cells**. *Mol Cell* 2001, **7**(3):673-682.

273. Yu PW, Huang BC, Shen M, Quast J, Chan E, Xu X, Nolan GP, Payan DG, Luo Y: **Identification of RIP3, a RIP-like kinase that activates apoptosis and NFkappaB**. *Curr Biol* 1999, **9**(10):539-542.

274. Yu T, Wang X, Purring-Koch C, Wei Y, McLendon GL: **A mutational epitope for cytochrome C binding to the apoptosis protease activation factor-1**. *J Biol Chem* 2001, **276**(16):13034-13038.

275. Yuste VJ, Sanchez-Lopez I, Sole C, Moubarak RS, Bayascas JR, Dolcet X, Encinas M, Susin SA, Comella JX: **The contribution of apoptosis-inducing factor, caspase-activated DNase, and inhibitor of caspase-activated DNase to the nuclear phenotype and DNA degradation during apoptosis**. *J Biol Chem* 2005, **280**(42):35670-35683.

276. Zha J, Harada H, Osipov K, Jockel J, Waksman G, Korsmeyer SJ: **BH3 domain of BAD is required for heterodimerization with BCL-XL and pro-apoptotic activity**. *J Biol Chem* 1997, **272**(39):24101-24104.

277. Zhang, Wang H: **MDM2 oncogene as a novel target for human cancer therapy**. *Curr Pharm Des* 2000, **6**(4):393-416.

278. Zhang G, Ghosh S: **Negative regulation of toll-like receptor-mediated signaling by Tollip**. *J Biol Chem* 2002, **277**(9):7059-7065.

279. Zhang XM, Weber I, Chen MJ: **Site-directed mutational analysis of human tumor necrosis factor-alpha receptor binding site and structure-functional relationship**. *J Biol Chem* 1992, **267**(33):24069-24075.

280. Zhang Y, Dimtchev A, Dritschilo A, Jung M: **Ionizing radiation-induced apoptosis in ataxia-telangiectasia fibroblasts. Roles of caspase-9 and cellular inhibitor of apoptosis protein-1**. *J Biol Chem* 2001, **276**(31):28842-28848.

281. Zheng Y, Ouaaz F, Bruzzo P, Singh V, Gerondakis S, Beg AA: **NF-kappa B RelA (p65) is essential for TNF-alpha-induced fas expression but dispensable for both TCR-induced expression and activation-induced cell death**. *J Immunol* 2001, **166**(8):4949-4957.

282. Zou T, Rao JN, Guo X, Liu L, Zhang HM, Strauch ED, Bass BL, Wang JY: **NF-kappaB-mediated IAP expression induces resistance of intestinal epithelial cells to apoptosis after polyamine depletion**. *Am J Physiol Cell Physiol* 2004, **286**(5):C1009-1018.
