# Supplementary material for: A logic-based diagram of signalling pathways central to macrophage activation
Source: BMC Syst Biol. 2008 Apr 23;2:36. doi: 10.1186/1752-0509-2-36 (PMC2383880; doi:10.1186/1752-0509-2-36)
Supplement: Additional file 4 — Description of the biological content of the pathway. A description of the signalling of the four pathways (Toll-like receptor, interferon, NF-κB and apoptosis) depicted on the integrated pathway diagram is provided here. The interconnectivity of these pathways and their significance in innate immune signalling is also discussed in this section. [file 1752-0509-2-36-S4.doc]

## Biological Content of Pathway Map

An organism’s first line of defence against invasion by pathogens is the ability to detect non-self material so a tailored immune response can be elicited. Recognition of ‘foreign’ entities is primarily performed by pattern recognition receptors (PRRs) of which toll-like receptors (TLR’s) are the best-characterised family. The function of TLR’s is to trigger the immune response by activation of the NF-κB and IRF (interferon regulatory factor) signalling pathways. We have included eight of the 11 reported mammalian TLR’s on the current map. Residing in the cell membrane (TLR’s 1,2,4 and 6) or endosomal compartments (3,7 and 9) [1] TLR’s comprise two functionally significant domains; one for recognising specific pathogen associated molecular patterns (PAMPs) and one for recruiting signalling adaptor proteins once the receptors are stimulated by binding of an appropriate pathogen-derived ligand. Whilst the pathogen recognition domains of different TLR’s are highly variable structurally [2], thereby allowing the recognition of diverse pathogen material ranging from viral double- and single-stranded RNA [3, 4] bacterial flagellin [5, 6], lipopeptides [7, 8], lipopolysaccharides [9, 10], and bacterial and viral CpG motifs [11, 12], the internal domains tend to be more conserved, reflecting the ability of different TLR’s to recruit the same adaptor proteins. For example a key adaptor protein common between TLR’s 1,2,5,6,7 and 9 is MYD88 (myeloid differentiation primary response gene 88). MYD88 is well documented to link TLR signalling to the NF-κB pathway [13-15]. A comprehensive and systematic effort to depict TLR signalling has recently been reported elsewhere [16] and in comparison the current effort is perhaps a rather simplistic one, if somewhat easier to follow, view of events. Although the known associations between TLR signalling and the MAP-kinase, ERK and JNK pathways [17] are yet to be included we have depicted the initial activation of MAP-kinases and the concomitant activation of the NF-κB pathway.

One of the major holes in our understanding of pathway architecture is transcriptional activation. Here we have attempted to display some of what is known about the gene targets of these pathways but there is clearly much missing from these views both in terms of the complexity of the transcription machinery and the genes activated. What is clear however is that TLR signalling directly leads to the activation of interferon signalling [18] which plays a central role in co-ordinating many aspects the innate immune response. Interferons represent a family of secreted cytokines and are often described as being either type I or type II. The latter referring to signalling via the IFNγ receptor (IFNGR), stimulated by binding of the IFNγ (IFNG), a cytokine synthesised by activated T-lymphocyte and natural killer cells in recognition of infected cells. Type I interferon signalling is co-ordinated by IFNα and IFNβ receptors (IFNAR/IFNBR) which are activated by their respective interferons in direct response to infection [19]. Ligand binding to both subsets of receptors induces a phosphorylation cascade eventually resulting in the activation of transcription factors STAT1, STAT2 and members of the IRF family of protein. This in turn leads to the transcriptional activation of many of the genes involved in immune and cellular defence processes, only some of which we have been able to directly link to the activity of specific transcription factors. However, it is clear that these include key signalling molecules (STAT1, IRF2), activating ligands of the apoptosis pathway (FASLG, TNFSF10), cytokines (IL1B, IL15, IL12B, CCL5, CXCL9), proteins involved in antigen presentation (C2TA – a key regulator of class II molecules, PSMB subunits, TAP1), cell adhesion (ICAM1) and a whole range of interferon responsive genes many of which are still of unknown function [20, 21].

NF-κB signalling is activated in response to numerous stress signals and is essential in orchestrating the immune response. We have described the activation of three different homo and heterodimer NF-κB complexes (although others do exist) [22]. Activation of RELA/NFKB1 complex is commonly referred to as the canonical NF-κB pathway and is generally associated with promoting a pro-apoptotic response by modulating expression of specific genes. Signalling incorporating RELB and NFKB2 proteins is often termed the alternative NF-κB pathway and is associated with cell survival. Hence apoptosis is a carefully regulated process and ultimately the innate immune response may culminate in host cell suicide thereby potentially limiting further reproduction of pathogenic organisms such as viruses.

Two major routes of apoptosis execution have been identified; termed intrinsic and extrinsic pathways. The intrinsic pathway is activated as a result of stress signals detected within the cell, for example, penetration of a viral pathogen into the cell or UV light induced DNA damage. Extrinsic apoptosis on the other hand is triggered by extracellular death-signalling ligands (FAS, TNFSF10 (TRAIL), TNF) binding to the cell membrane receptors. Both intrinsic and extrinsic pathways activate a number of the caspase family of cysteine proteases. The initial caspases to be activated are categorised as initiators,(CASP’s 1,2,4,6,8,9,10) and are capable of cleaving downstream executioner caspases, specifically CASP3 and CASP7, so called as they are directly responsible for morphological changes in the cell associated with apoptosis by the cleavage or inactivation of an array of molecules including, structural proteins, DNA repair proteins, and anti-apoptotic proteins.

Supplementary Text Bibliography

1. Nishiya T, DeFranco AL: **Ligand-regulated chimeric receptor approach reveals distinctive subcellular localization and signaling properties of the Toll-like receptors**. *J Biol Chem* 2004, **279**(18):19008-19017.

2. Kirk P, Bazan JF: **Pathogen recognition: TLRs throw us a curve**. *Immunity* 2005, **23**(4):347-350.

3. Alexopoulou L, Holt AC, Medzhitov R, Flavell RA: **Recognition of double-stranded RNA and activation of NF-kappaB by Toll-like receptor 3**. *Nature* 2001, **413**(6857):732-738.

4. Lund JM, Alexopoulou L, Sato A, Karow M, Adams NC, Gale NW, Iwasaki A, Flavell RA: **Recognition of single-stranded RNA viruses by Toll-like receptor 7**. *Proc Natl Acad Sci U S A* 2004, **101**(15):5598-5603.

5. Feuillet V, Medjane S, Mondor I, Demaria O, Pagni PP, Galan JE, Flavell RA, Alexopoulou L: **Involvement of Toll-like receptor 5 in the recognition of flagellated bacteria**. *Proc Natl Acad Sci U S A* 2006, **103**(33):12487-12492.

6. Hayashi F, Smith KD, Ozinsky A, Hawn TR, Yi EC, Goodlett DR, Eng JK, Akira S, Underhill DM, Aderem A: **The innate immune response to bacterial flagellin is mediated by Toll-like receptor 5**. *Nature* 2001, **410**(6832):1099-1103.

7. Nakao Y, Funami K, Kikkawa S, Taniguchi M, Nishiguchi M, Fukumori Y, Seya T, Matsumoto M: **Surface-expressed TLR6 participates in the recognition of diacylated lipopeptide and peptidoglycan in human cells**. *J Immunol* 2005, **174**(3):1566-1573.

8. Takeda K, Takeuchi O, Akira S: **Recognition of lipopeptides by Toll-like receptors**. *J Endotoxin Res* 2002, **8**(6):459-463.

9. Arbour NC, Lorenz E, Schutte BC, Zabner J, Kline JN, Jones M, Frees K, Watt JL, Schwartz DA: **TLR4 mutations are associated with endotoxin hyporesponsiveness in humans**. *Nat Genet* 2000, **25**(2):187-191.

10. Rhee SH, Hwang D: **Murine TOLL-like receptor 4 confers lipopolysaccharide responsiveness as determined by activation of NF kappa B and expression of the inducible cyclooxygenase**. *J Biol Chem* 2000, **275**(44):34035-34040.

11. Lamphier MS, Sirois CM, Verma A, Golenbock DT, Latz E: **TLR9 and the recognition of self and non-self nucleic acids**. *Ann N Y Acad Sci* 2006, **1082**:31-43.

12. Takeshita F, Leifer CA, Gursel I, Ishii KJ, Takeshita S, Gursel M, Klinman DM: **Cutting edge: Role of Toll-like receptor 9 in CpG DNA-induced activation of human cells**. *J Immunol* 2001, **167**(7):3555-3558.

13. Aliprantis AO, Yang RB, Weiss DS, Godowski P, Zychlinsky A: **The apoptotic signaling pathway activated by Toll-like receptor-2**. *Embo J* 2000, **19**(13):3325-3336.

14. Medzhitov R, Preston-Hurlburt P, Kopp E, Stadlen A, Chen C, Ghosh S, Janeway CA, Jr.: **MyD88 is an adaptor protein in the hToll/IL-1 receptor family signaling pathways**. *Mol Cell* 1998, **2**(2):253-258.

15. Schnare M, Holt AC, Takeda K, Akira S, Medzhitov R: **Recognition of CpG DNA is mediated by signaling pathways dependent on the adaptor protein MyD88**. *Curr Biol* 2000, **10**(18):1139-1142.

16. Oda K, Kitano H: **A comprehensive map of the toll-like receptor signaling network**. *Mol Syst Biol* 2006, **2**:2006 0015.

17. Dong C, Davis RJ, Flavell RA: **MAP kinases in the immune response**. *Annu Rev Immunol* 2002, **20**:55-72.

18. Colonna M: **TLR pathways and IFN-regulatory factors: to each its own**. *Eur J Immunol* 2007, **37**(2):306-309.

19. Goodbourn S, Didcock L, Randall RE: **Interferons: cell signalling, immune modulation, antiviral response and virus countermeasures**. *J Gen Virol* 2000, **81**(Pt 10):2341-2364.

20. de Veer MJ, Holko M, Frevel M, Walker E, Der S, Paranjape JM, Silverman RH, Williams BR: **Functional classification of interferon-stimulated genes identified using microarrays**. *J Leukoc Biol* 2001, **69**(6):912-920.

21. Indraccolo S, Pfeffer U, Minuzzo S, Esposito G, Roni V, Mandruzzato S, Ferrari N, Anfosso L, Dell'Eva R, Noonan DM *et al*: **Identification of genes selectively regulated by IFNs in endothelial cells**. *J Immunol* 2007, **178**(2):1122-1135.

22. Ghosh S, May MJ, Kopp EB: **NF-kappa B and Rel proteins: evolutionarily conserved mediators of immune responses**. *Annu Rev Immunol* 1998, **16**:225-260.
